# Impact of Pharmacist-Led Interventions on Patient Outcomes in Gulf Cooperation Council Countries: A Systematic Review and Meta-Analysis

**DOI:** 10.3390/pharmacy14040102

**Published:** 2026-07-06

**Authors:** Saleh Alghamdi

**Affiliations:** Department of Clinical Pharmacy, Faculty of Pharmacy, Al-Baha University, Al-Baha 65779, Saudi Arabia; saleh.alghamdi@bu.edu.sa

**Keywords:** pharmacist-led interventions, clinical pharmacy, gulf cooperation council (GCC) countries, patient outcomes, medication adherence, systematic review, meta-analysis

## Abstract

Background: Pharmacist-led interventions in Gulf Cooperation Council (GCC) countries have been increasingly studied, yet their overall effectiveness across clinical and healthcare outcomes remains incompletely defined. To address this gap, a systematic review and meta-analysis of randomized controlled trials and quasi-experimental studies published between 2000 and 2025 was conducted, following PRISMA 2020 and Cochrane standards. Methods: Searches of PubMed/MEDLINE, Scopus, Web of Science, and CENTRAL identified 437 records, of which 20 studies met the inclusion criteria; 13 contributed meta-analyzable data as randomized controlled trials and seven as quasi-experimental studies. Results: Pooled random-effects estimates favored pharmacist-led care for HbA1c, fasting glucose, low-density lipoprotein cholesterol, diastolic blood pressure, and medication knowledge, although these estimates carried substantial, largely unexplained heterogeneity and were rated as low to very low certainty under GRADE. The effects on systolic blood pressure, total cholesterol, triglycerides, high-density lipoprotein cholesterol, unplanned healthcare use, and antimicrobial utilization were favorable but not statistically significant. Quasi-experimental studies consistently demonstrated reductions in mortality and readmissions, though hospital and ICU length of stay remained variable. Risk of bias was judged as some concerns for randomized trials and moderate to serious for quasi-experimental studies, with substantial heterogeneity observed across blood pressure and lipid outcomes. Conclusions: Overall, pharmacist-led interventions in GCC settings were associated with improvements in glycemic control and LDL cholesterol, with additional benefits in mortality and readmissions, although the certainty of evidence was low to very low, owing to substantial heterogeneity and the predominance of non-randomized designs for the inpatient outcomes. These findings underscore the need for standardized intervention models and outcome measures.

## 1. Introduction

The role of pharmacists has evolved from product-focused dispensing toward patient-centered clinical care, emphasizing medication safety, therapeutic optimization, and integration within multidisciplinary healthcare teams. This shift is increasingly evident in GCC countries, including Saudi Arabia, the United Arab Emirates (UAE), Kuwait, Qatar, Oman, and Bahrain, where healthcare systems are undergoing substantial transformation [1]. National strategies such as Saudi Arabia’s Vision 2030 and the UAE National Health Strategy 2024 highlight the expanding role of pharmacists in delivering integrated, patient-centered care within multidisciplinary settings [2].

Evidence from international studies indicates that pharmacist-led interventions in chronic disease management improve glycemic and cardiovascular outcomes, enhance medication adherence and disease understanding, and reduce medication-related complications and preventable hospital readmissions [3,4,5]. In infectious disease settings, pharmacist involvement in antimicrobial stewardship programs has been associated with reductions in inappropriate antibiotic use and healthcare costs [6]. These findings suggest that the contribution of clinical pharmacists extends beyond dispensing to improving patient outcomes and healthcare efficiency. Despite the growing body of literature, evidence specific to GCC country settings remains limited and fragmented. Differences in healthcare systems, practice models, accreditation standards, and workforce development may limit the generalizability of international findings to the region [7]. Moreover, the period from 2000 to 2025 represents a phase of expansion in clinical pharmacy services in the GCC countries, characterized by the introduction of pilot programs and policy-driven integration into healthcare systems [1].

This study systematically reviews and meta-analyzes the effectiveness of pharmacist-led interventions in GCC countries compared with usual care, focusing on clinical, humanistic, and economic outcomes, and providing a comprehensive regional synthesis across multiple outcome domains.

## 2. Materials and Methods

### 2.1. Search Strategy

This review adhered to the Preferred Reporting Items for Systematic Review and Meta-Analysis (PRISMA) 2020 framework and followed the methodological standards outlined in the Cochrane Handbook for Systematic Reviews of Interventions [8,9,10]. The review protocol was registered prospectively with the National Institute of Health’s PROSPERO database (Registration ID: CRD420261358069). Eligibility criteria and outcomes were predefined using the PICOS framework [11]. A comprehensive electronic search was conducted from January 2000 to December 2025 in Scopus, PubMed/MEDLINE, Web of Science, and the Cochrane Central Register of Controlled Trials (CENTRAL). The search strategy incorporated both MeSH terms and free-text keywords related to pharmacist-provided services such as pharmacist intervention, clinical pharmacy services, pharmacist-led care, medication therapy management, medication reconciliation, and pharmaceutical care, combined with geographic identifiers for GCC countries, including Saudi Arabia, United Arab Emirates, Qatar, Oman, Kuwait, and Bahrain (Supplementary File S3). Search strategies were adapted for each database, and additional papers were searched from the reference lists of studies included.

### 2.2. Eligibility Criteria

Controlled quasi-experimental studies and randomized controlled trials (RCTs) were included, along with controlled before-and-after designs and non-randomized controlled trials, as recommended by guidelines [8]. Interventions were defined as pharmacist-led services beyond routine dispensing, including medication therapy management, medication reconciliation, clinical review, disease education, antimicrobial stewardship (AMS), and multidisciplinary care [12,13,14,15]. Eligible studies required a comparator group and at least one quantitative outcome within predefined categories: glycemic control (primary endpoint: HbA1c reduction), secondary clinical outcomes (blood pressure and lipid profile), humanistic outcomes (medication adherence and quality of life), and healthcare utilization (hospital readmissions). Studies conducted outside GCC countries or lacking a comparator were excluded, as were descriptive designs (cross-sectional, qualitative, single-arm pre–post, conference abstracts without full data, reviews, editorials, and case reports). This hierarchical outcome framework ensured consistency and relevance to both clinical practice and national health priorities.

### 2.3. Screening Process and Data Extraction

Screening was completed by two reviewers (S.A. and M.A). Each reviewer independently reviewed titles, abstracts, and, subsequently, full texts. Any discrepancies were clarified through reviewer discussion. Data extraction and analysis were performed by S.A. and independently verified by M.A. Records were managed using Microsoft ® Excel for deduplication, screening, and documentation [16]. The database supported de-duplication, eligibility tracking, and exclusion documentation (Supplementary File S1). The data extraction used a standardized Excel form, capturing (1) first author and publication year, (2) study location (country), (3) study design, (4) intervention setting, (5) study population, (6) sample size (intervention/control), (7) pharmacists’ intervention, (8) comparator, and (9) measured outcomes, as shown in Table 1 and Table 2.

### 2.4. Meta-Analysis Methods and Heterogeneity Assessment

For outcomes with at least three comparable studies, estimates were generated using meta-analytic techniques; when fewer studies were available, the results were summarized narratively. All analyses were conducted in R (version 4.5.2) through RStudio (version 2026.01.1). Customized R code was developed with the assistance of generative AI tools (Microsoft Copilot (April 2026 version) and Google AI (April 2026 version), to support meta-analytic plotting, sensitivity analyses, and data visualization. The generated code was reviewed, validated, and adapted by the author to ensure accuracy, reproducibility, and alignment with Cochrane methodological standards [37,38,39,40]. Continuous variables were synthesized using either mean differences or standardized mean differences, while dichotomous outcomes were expressed as risk ratios, each accompanied by 95% confidence intervals. To maintain the highest level of internal validity and scientific rigor, we implemented a pre-specified tiered evidence synthesis framework. RCTs were designated as the primary body of evidence for estimating relative treatment effects due to their inherent protection against confounding. Non-randomized/quasi-experimental studies were analyzed as a strictly separate, complementary dataset to provide insight into real-world clinical effectiveness and long-term outcomes in the GCC region. Crucially, no ‘grand’ pooled effect size was calculated across these disparate study designs. All meta-analytic estimates were generated within isolated design-specific subgroups (RCT vs. quasi-experimental) to prevent selection bias and the artificial inflation of sample sizes. To evaluate variability across studies, heterogeneity was quantified using the I^2^ and τ^2^ statistics. As a general guide, I^2^ values near 25%, 50%, and 75% were interpreted as low, moderate, and high heterogeneity. Because I^2^ is a relative measure that is inflated when the pooled trials are individually large and precise, it was interpreted alongside τ^2^ and the prediction interval rather than as an absolute measure of incompatibility between studies [41,42,43]. For every outcome pooled from at least three studies, a 95% prediction interval was calculated in addition to the confidence interval of the mean to express the range of true effects expected in a new setting [44]. The certainty of evidence for each outcome was rated using the GRADE approach, with downgrading for risk of bias, inconsistency, and imprecision [45,46,47]. Randomized evidence began at high certainty and non-randomized evidence at low certainty, with the latter rated in accordance with GRADE guidance for studies assessed with ROBINS-I [48]. The resulting per-outcome ratings are summarized in Table 3. The robustness of pooled estimates was examined through leave-one-out sensitivity analyses. When an adequate number of studies were available, publication bias was explored using funnel plots and Egger’s regression test, with statistical significance set at *p* < 0.05 [49,50]. In addition, trim-and-fill analysis was performed to estimate the potential impact of missing studies on pooled effect sizes, providing a sensitivity check for small-study effects and possible overestimation. Additional analyses are presented in Supplementary File S2.

### 2.5. Risk-of-Bias Assessment

**Figure 1:**
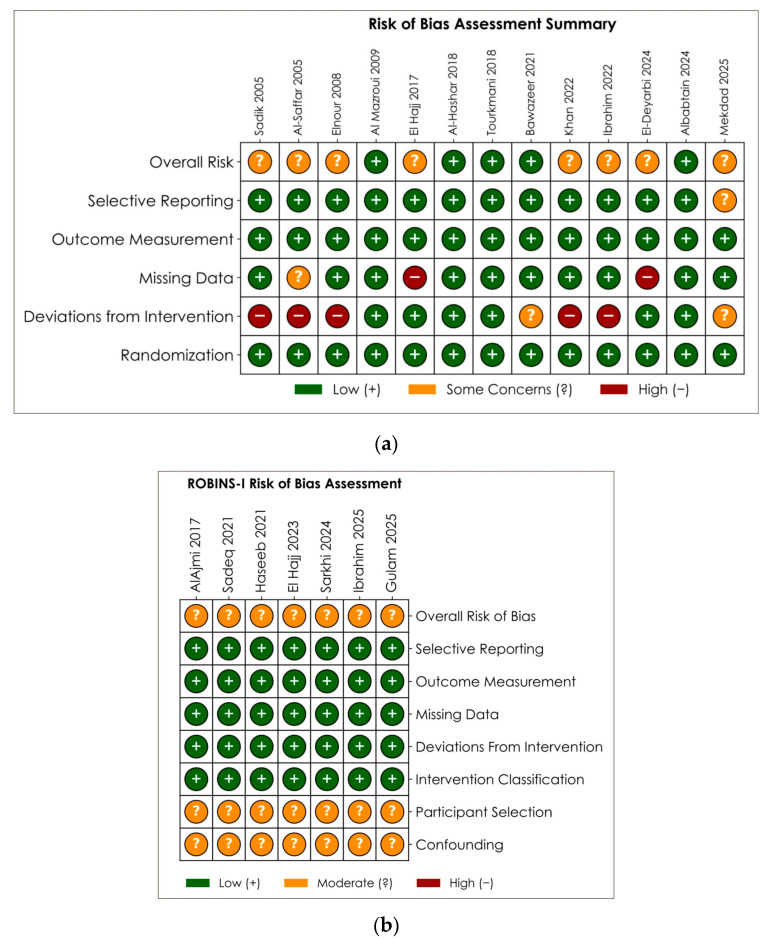
Risk-of-bias summary. (**a**) RoB 2 traffic light and weighted bar plots for the 13 randomized controlled trials across the five RoB 2 domains and the overall judgment. Green denotes low risk, yellow some concerns or moderate risk, and red serious risk [31,32,33,34,35,36,37,38,39,40,41,42,43]. (**b**) ROBINS-I assessment of the seven non-randomized controlled studies across the seven ROBINS-I domains and the overall judgment. labels: green—low risk, yellow—some concerns or moderate risk, and red—serious risk [44,45,46,47,48,49,50].

RCTs were assessed using the RoB 2 tool [51], and controlled quasi-experimental studies were evaluated using the ROBINS-I tool [52]. Two reviewers (S.A. and M.A.) independently performed assessments, with disagreements resolved by consensus. The summary judgments and domain-level assessments are reported in Figure 1a,b. The details can be seen in Supplementary File S3.

## 3. Results

### 3.1. Study Selection

**Figure 2:**
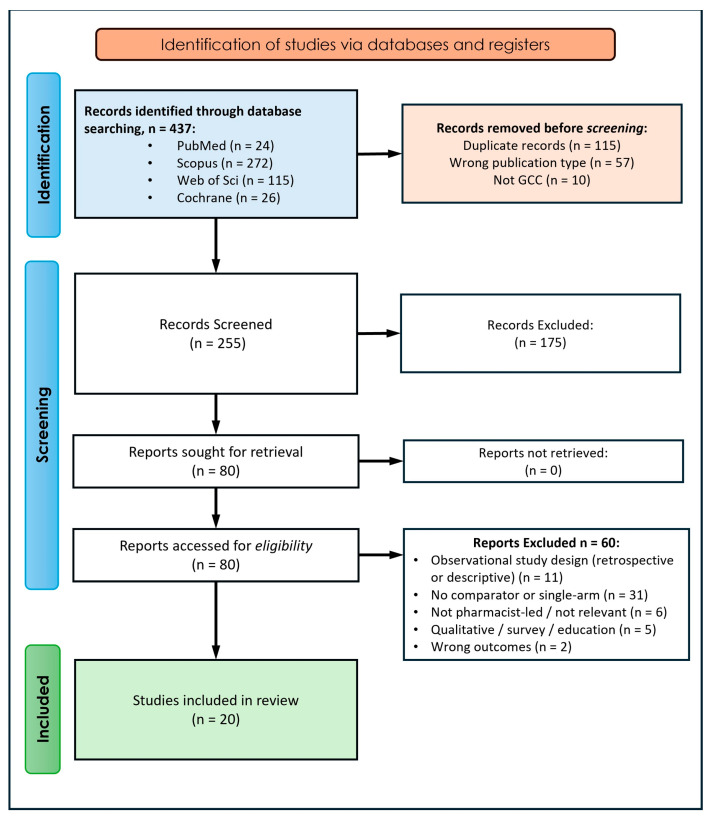
PRISMA diagram describing study selection process.

The database searches identified 437 records. After removing 182 duplicates and ineligible records, 255 were retained for title and abstract screening. Of these, 175 were excluded. Eighty full-text articles were assessed, and 60 were excluded for reasons such as observational design, absence of a comparator, non-pharmacist-led interventions, qualitative design, or irrelevant outcomes. Ultimately, 20 studies were included in the systematic review and meta-analysis, as shown in PRISMA Figure 2. Among the included studies, 11 were parallel-group randomized controlled trials, while two employed alternative randomized designs (an open three-arm RCT and a prospective randomized pre–post design). For analysis, all were grouped under RCTs, but sensitivity checks considered design differences. Seven were prospective non-randomized controlled studies. To minimize bias from design heterogeneity, RCTs were analyzed separately from non-randomized controlled studies.

### 3.2. Characteristics of Included Studies

(a) Randomized studies

Thirteen randomized trials (3754 participants) were included in the primary meta-analysis presented in Table 1. The studies were conducted in Saudi Arabia (*n* = 5), the UAE (*n* = 5), Oman (*n* = 1), Qatar (*n* = 1), and Kuwait (*n* = 1) across community pharmacies, inpatient wards, outpatient clinics, and ambulatory centers. Most trials evaluated pharmacist-led interventions targeting chronic disease management, particularly type 2 diabetes, which represented the largest study group [20,23,25,27,28,29]. Other clinical areas included hypertension [26], heart failure [17], smoking cessation [21], epilepsy [18], depression [18], gestational diabetes [19], and medication safety during transitions of care [22,24]. Interventions typically comprised medication therapy management, reconciliation, pharmaceutical care programs, structured education/counselling, telepharmacy, and multidisciplinary care, whereas comparators received usual care. Across the included trials, the outcomes were primarily clinical, including glycemic control (HbA1c) [19,20,23,25,27,28,29], fasting blood glucose (FBG) [19,20,23,29], blood pressure [17,20,23,26,28], and lipid profile [20,23,25,28,29], and humanistic, such as medication adherence [17,18,20,27], adequate medication knowledge [17,18,20], and SF-36 quality-of-life indicators [17,19,20].

(b) Quasi-Experimental Studies

Seven quasi-experimental/non-randomized controlled studies (4631 participants) were included in the secondary analysis (Table 2). The studies were from Saudi Arabia (*n* = 4), the UAE (*n* = 2), and Qatar (*n* = 1) and were mainly hospital-based (inpatient wards and ICUs), with one outpatient neurology clinic [30]. Most evaluated pharmacist-led antimicrobial stewardship, prospective audit and feedback, multidisciplinary review, and guideline implementation [31,32,34,36]. Other interventions included pharmacist independent prescribing in critical care [35], structured discharge counseling with medication reconciliation after acute coronary syndromes [33], and pharmacist-led educational interviews to improve adherence in epilepsy [30]. Comparators were typically pre-intervention phases or usual care. The most frequently reported outcomes were clinical and healthcare utilization measures, including antibiotic utilization metrics such as defined daily dose (DDD) and days of therapy (DOTs) [31,36], hospital and ICU length of stay [34,35], hospital readmissions [31,34,36], and mortality [31,32,33,34,35,36]. Some studies also assessed economic outcomes, such as cost-effectiveness, and humanistic outcomes, including medication adherence [30].

### 3.3. Publication Bias Analysis

Funnel plots and Egger’s tests showed visual asymmetry for HbA1c and systolic BP (Egger non-significant), borderline small-study effects for fasting glucose, and modest asymmetry for diastolic BP. Given the few studies and substantial heterogeneity, the power to detect bias was low. Plots for all the other parameters can be observed in detail in Supplementary File S2.

### 3.4. Results: Randomized Controlled Trials (RCTs)

#### 3.4.1. Clinical Outcomes

##### HbA1c Levels (Primary Outcome)

**Figure 3:**
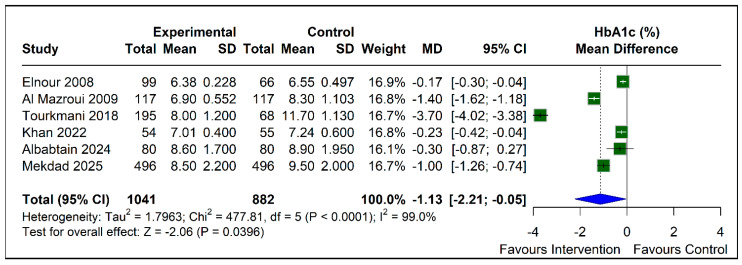
Forest plot of HbA1c (%) mean differences between pharmacist-led intervention and usual-care control groups across six randomized controlled trials (random-effects model, k = 6, and I^2^ = 99%). Each green square represents the mean difference estimate from an individual study, with the square size proportional to its statistical weight. The horizontal line through each square indicates the 95% confidence interval (CI). The blue diamond at the bottom represents the pooled overall effect size, with its width corresponding to the 95% CI. Measures of heterogeneity (Tau^2^, Chi^2^, I^2^) and the overall effect test are reported below the plot [19,20,23,25,28,29].

Across randomized controlled trials, pharmacist-led interventions improved glycemic and outcomes versus usual care, as shown in Figure 3. Pooling six RCTs (*n* = 1.923), pharmacist-led care significantly reduced HbA1c [MD = −1.13% (95% CI: −2.21 to −0.05) and *p* = 0.0396)] with very high heterogeneity (I^2^ = 99%; *p* < 0.0001). The corresponding 95% prediction interval was −4.50 to 2.23%, which, by crossing zero, indicates that the true effect in a new setting could plausibly include little or no benefit. Given the very high heterogeneity, this outcome was rated as very low certainty under GRADE (Table 3) despite the nominally significant pooled estimate.

##### HbA1c Sub-Group Analysis

**Figure 4:**
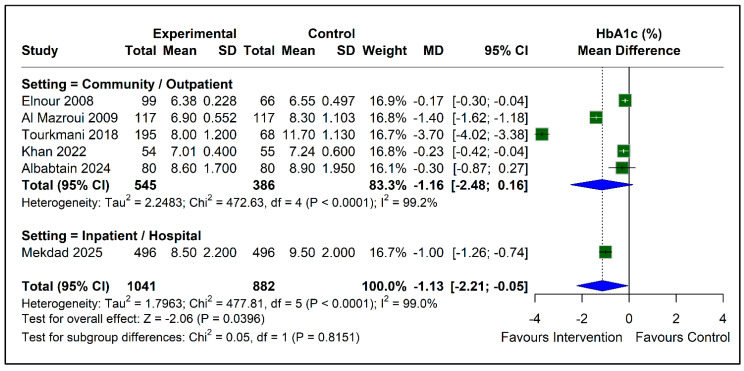
Subgroup forest plot of HbA1c mean differences between intervention and control groups, stratified by outpatient (community) and inpatient (hospital) settings, with pooled effect sizes and 95% confidence intervals [19,20,23,25,28].

Pre-specified subgroup analyses were conducted to examine whether the clinical setting or country of study origin contributed to the observed heterogeneity in HbA1c outcomes. When studies were stratified by clinical setting, the pooled MD was −1.16% (95% CI: −2.48 to 0.16) for community and outpatient studies (k = 5) and −1.00% (95% CI: −1.26 to −0.74) for the single inpatient study [29]. The test for subgroup differences was non-significant (χ^2^ = 0.05, df = 1, and *p* = 0.815), indicating that the clinical setting did not account for the observed heterogeneity and that the direction and magnitude of effect were consistent across both contexts, as described in Figure 4.

**Figure 5:**
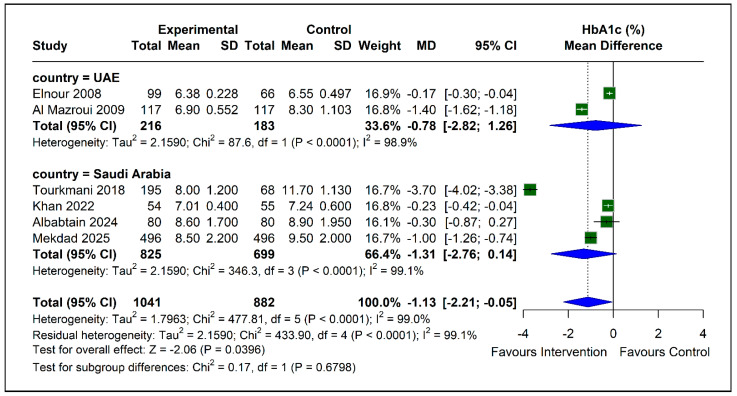
Subgroup forest plot of HbA1c mean differences between intervention and control groups, stratified by country (UAE vs. Saudi Arabia) [19,20,23,25,28,29].

When stratified by country, the pooled MD was −0.78% (95% CI: −1.99 to 0.42) for UAE studies (k = 2) and −1.31% (95% CI: −2.91 to 0.29) for Saudi Arabia studies (k = 4), with a non-significant test for subgroup differences (χ^2^ = 0.27, df = 1, *p* = 0.605). In summary, these analyses suggest that the heterogeneity in HbA1c outcomes reflects variability in intervention intensity and baseline glycemic control across individual studies rather than systematic differences attributable to setting or country, as shown in Figure 5.

##### Publication Bias Analysis for HbA1c

**Figure 6:**
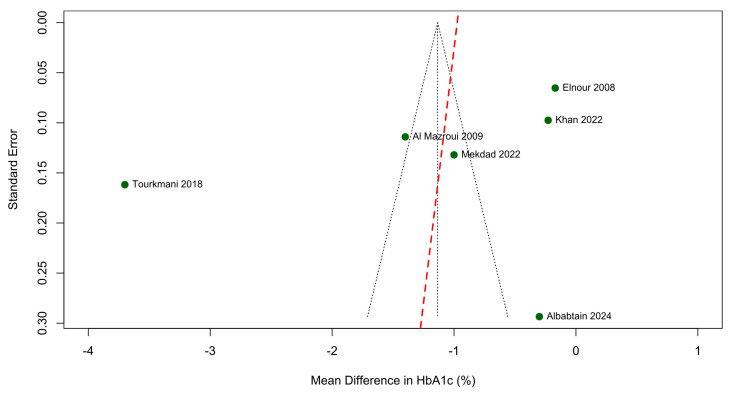
Funnel plot of HbA1c mean differences showing study precision and effect size distribution. Each green dot represents an individual study plotted by its mean difference and standard error. The red dashed vertical line indicates the overall pooled effect estimate. The black dotted diagonal lines form the expected 95% confidence region (funnel shape). The distribution shows some asymmetry, indicating potential small study effects or publication bias, though not extreme [19,20,23,25,28,29].

The funnel plot is shown in Figure 6. To further evaluate the robustness of pooled estimates in the presence of funnel plot asymmetry, a trim-and-fill analysis was performed for HbA1c as the primary outcome (Supplementary File S3, Figure S1). The algorithm imputed zero missing studies, indicating that the observed funnel plot asymmetry was not attributable to suppression of small negative studies. The adjusted pooled mean difference remained identical to the original estimate [MD = −1.13%; (95% CI: −2.21 to −0.05); *p* = 0.0396], supporting that the statistically significant HbA1c reduction was not solely attributable to publication bias. The Egger regression test was non-significant for HbA1c (*p* = 0.243), consistent with the trim-and-fill finding. Leave-one-out analyses produced pooled MDs from −1.33% to −0.63% (omission of Tourkmani et al.’s (2018) [23] study attenuated the effect and reduced the I^2^ to 96.4%) (Supplementary File S3, Figure S2); the overall RoB was low to moderate. Given the small number of studies (k = 6), both tests are acknowledged to be underpowered, and these results should be interpreted as supportive rather than definitive evidence against publication bias.

##### Fasting Blood Glucose (FBG)

**Figure 7:**
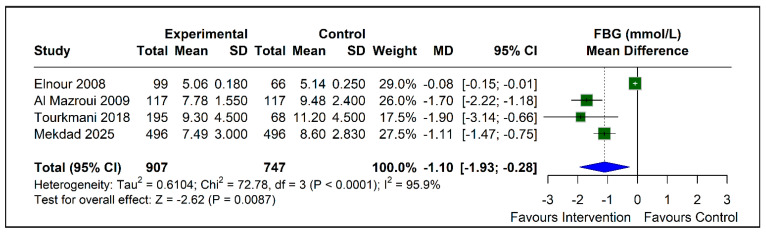
Forest plot of FBG (mmol/L) mean differences between intervention and control groups [19,20,23,29].

In the case of fasting blood glucose, pooling four RCTs significantly reduced the FBG [(MD = −1.10 mmol/L (95% CI: −1.93, −0.28) and *p*-value 0.0087)] (Figure 7). Although heterogeneity was high (I^2^ > 95%), the direction of the effect remained consistent. The corresponding 95% prediction interval was −5.46 to 3.21 mmol/L, consistent with the very high heterogeneity. Leaving out Elnour’s (2008) study [19] produced the largest change [(MD = −1.44 mmol/L (95% CI: −1.94, − 0.94); *p* < 0.0001)], reducing I^2^ to 52.2%. The funnel plot shows asymmetry, with borderline Egger’s regression (*p* = 0.0598), asking for cautious interpretation (Supplementary File S2).

##### Blood Pressure Outcomes

**Figure 8:**
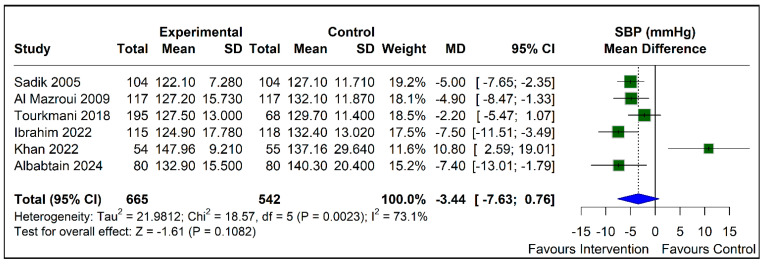
Forest plot of systolic blood pressure (mmHg) mean differences between intervention and control groups [17,20,23,25,26,28].

**Figure 9:**
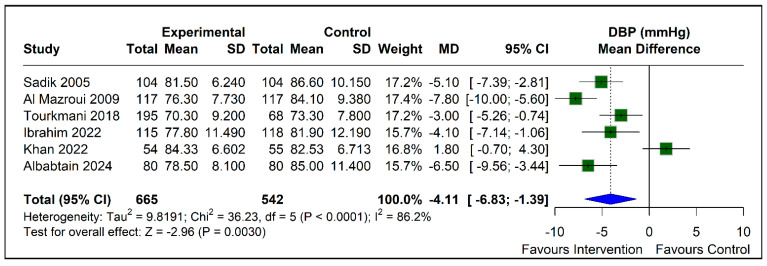
Forest plot of diastolic blood pressure (mmHg) mean differences between intervention and control groups [17,20,23,25,26,28].

Pooling six RCTs, the SBP showed a non-significant reduction (*p* = 0.1082) (Figure 8). The heterogeneity was moderate to high (I^2^ = 73.1%; *p* = 0.0023). Leave-one-out analysis identified Khan’s (2022) study [25] as influential, and its omission shifted the pooled estimate to −4.96 mmHg (*p* < 0.0001), reducing I^2^ to 20.1% (Supplementary File S2). The funnel plot suggested asymmetry, but Egger’s regression was non-significant (*p* = 0.3559). The corresponding 95% prediction interval was −13.90 to 6.43 mmHg. The DBP decreased across six RCTs [(MD = −4.11 mmHg (95% CI: −6.83, −1.39) and *p* = 0.003)], and the between-study heterogeneity was high (I^2^ = 86.2%). The most influential study was Khan’s (2022) [25]; excluding it gave MD = −5.31 mmHg (*p* < 0.0001) and I^2^ = 60.6%, as shown in Figure 9. The funnel plot shows mild asymmetry (Egger’s test; *p* = 0.3559 [verify: this Egger value is identical to the systolic estimate; insert the diastolic-specific value from metabias (m.dbp)]) (Supplementary File S2). The corresponding 95% prediction interval for DBP was −13.81 to 5.59 mmHg. Overall, the evidence suggests beneficial effects of pharmacist-led care on managing DBP, though the high heterogeneity warrants cautious interpretation. Egger’s test was performed, but it was underpowered with ≤7 studies, so its result is exploratory.

##### Lipid Profile Outcomes

**Figure 10:**
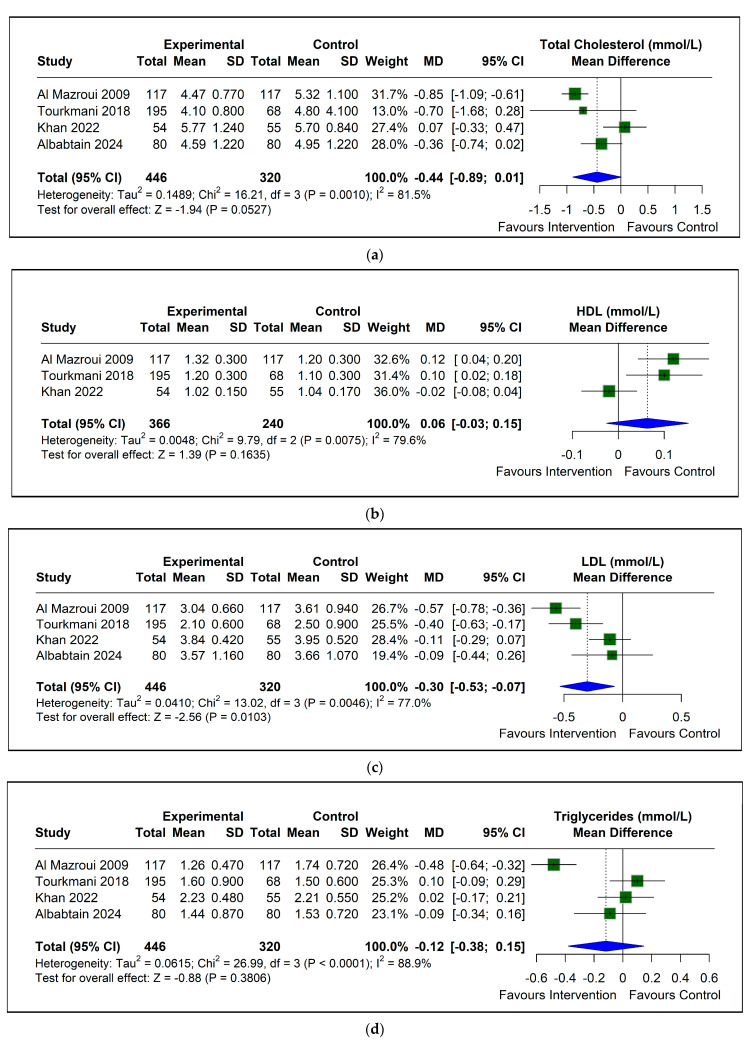
Forest plots of the mean differences for lipid parameters, including (**a**) total cholesterol, (**b**) LDL cholesterol, (**c**) HDL cholesterol, and (**d**) triglycerides [20,23,25,28].

Figure 10 presents the pooled effects of pharmacist-led interventions on lipid parameters, including (a) total cholesterol, (b) LDL cholesterol, (c) HDL cholesterol, and (d) triglycerides. Pharmacist-led interventions significantly reduced LDL cholesterol [MD = −0.30 mmol/L; 95% CI: −0.53 to −0.07; *p* = 0.0103]. The corresponding 95% prediction interval was −1.35 to 0.75 mmol/L. While changes in total cholesterol (*p* = 0.0527), HDL (*p* = 0.163), and triglycerides (*p* = 0.380) were directionally favorable, they did not reach statistical significance. Although the LDL reduction was statistically significant, the substantial between-study heterogeneity across lipid profiles (I^2^ ≈ 77–81.5%) and the borderline effect on total cholesterol warrant cautious interpretation.

#### 3.4.2. Humanistic Outcomes

##### Medication Adherence and Medication Knowledge

**Figure 11:**
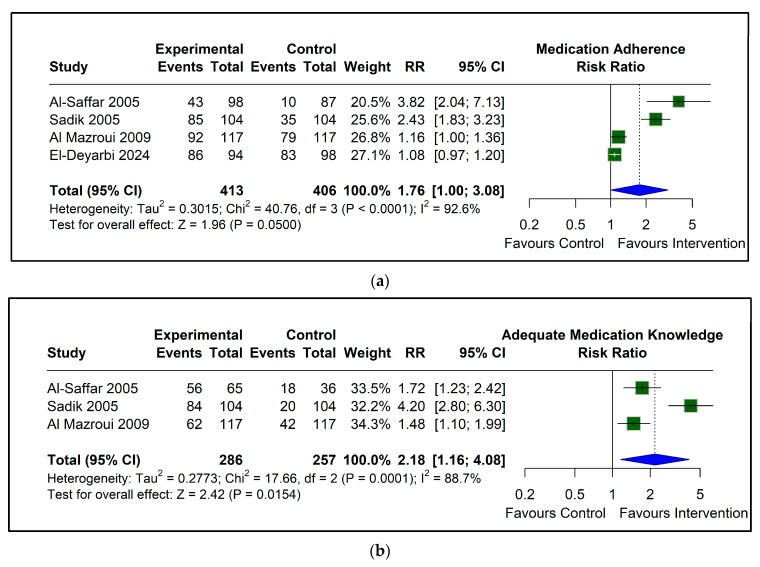
Forest plots of (**a**) medication adherence risk ratios and (**b**) adequate medication knowledge and pooled analyses [17,18,20,27].

The humanistic outcomes reported in randomized controlled trials focused on (a) medication adherence, and (b) adequate medication knowledge is shown in Figure 11. Pharmacist-led care demonstrated better outcomes in both analyses. Medication adherence improved marginally, reaching borderline significance (*p* = 0.050), while medication knowledge showed a statistically significant gain (*p* = 0.015). For medication adherence, the 95% prediction interval was wide (RR: 0.29 to 9.59), reflecting the high between-study heterogeneity. For medication knowledge, a stable prediction interval could not be estimated because only three studies were available, and it is, therefore, not reported. Both outcomes exhibited substantial heterogeneity (I^2^ = 92.6% and 88.7%, respectively), indicating considerable variability across trials. Overall, the results suggest a positive effect of pharmacist interventions on adherence and knowledge, although high heterogeneity limits the precision of pooled estimates.

##### SF 36 Quality-of-Life Domain Outcomes

All SF-36 domains showed statistically significant improvements favoring the intervention. The largest effects were seen for vitality, general health role (physical) and role (emotional), with mean differences ranging from roughly 9 to 21 points and consistently narrow confidence intervals (all *p* < 0.001). Physical functioning, bodily pain, social functioning, and mental health showed smaller but still statistically significant improvements, with mean differences generally between 9 and 14 points and wider confidence intervals (*p*-values from 0.0037 to 0.011). Heterogeneity was low for the domains with larger effects and substantial for those with smaller effects. Leave-one-out analyses indicated that the significant findings were robust across all domains. (Supplementary File S2, Figure S3).

### 3.5. Quasi-Experimental Studies

#### 3.5.1. Clinical Outcomes

##### Mortality, Readmissions, and Length of Stay

**Figure 12:**
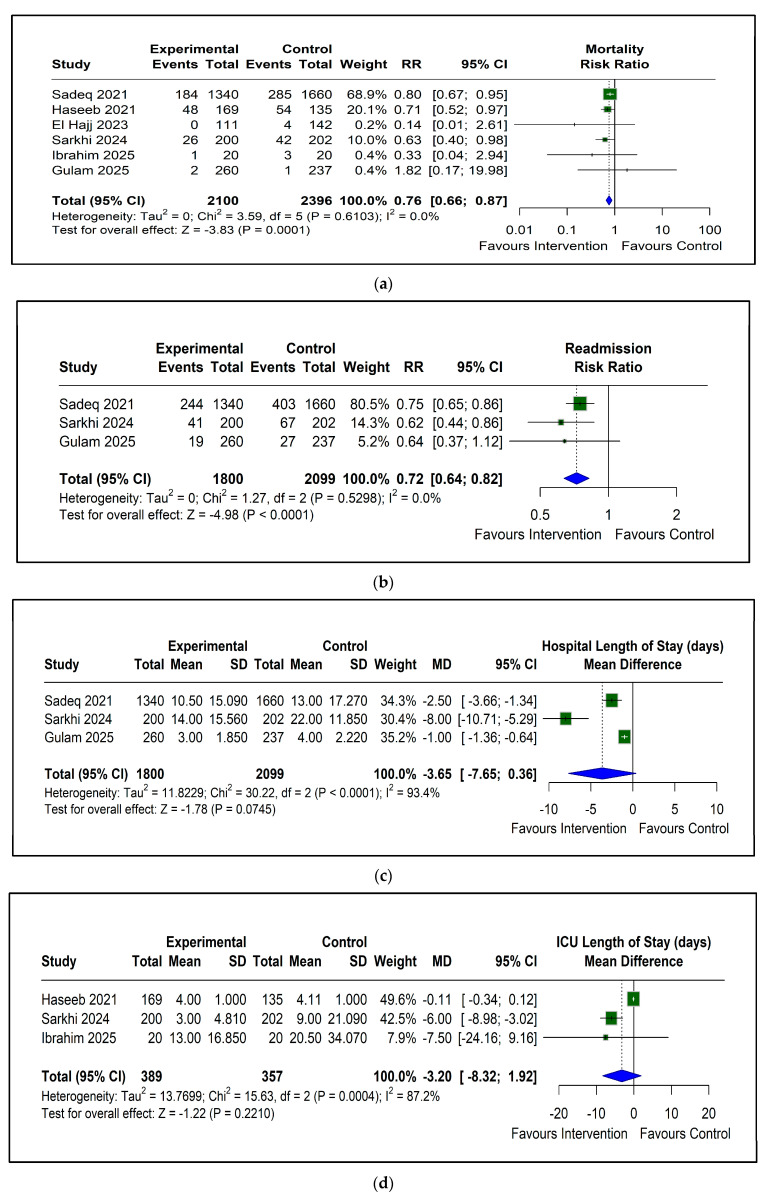
Forest plots of (**a**) mortality, (**b**) hospital readmissions, (**c**) hospital length of stay, and (**d**) ICU length of stay showing pooled analyses [31,32,33,34,35,36].

The main quasi-experimental clinical outcomes showed a mixed but clinically relevant pattern, as shown in Figure 12. Pharmacist-led interventions reduced (a) mortality (RR = 0.76; 95% CI: 0.66 to 0.87; *p* = 0.001) and (b) readmissions [(RR = 0.72 (95% CI: 0.64–0.82); *p* < 0.0001)], with no heterogeneity (I^2^ = 0%). The 95% prediction intervals were wide and crossed the null (RR: 0.54 to 1.07 for mortality and RR: 0.32 to 1.65 for readmissions), indicating that the true effect in a new setting is uncertain, and an I^2^ of 0% reflects agreement between estimates rather than freedom from bias. A sensitivity analysis excluding serious-risk studies was considered. On ROBINS-I, all six non-randomized studies contributing to the mortality and readmission pools were judged as moderate overall risk of bias; none of them was rated as serious. A restricted analysis would, therefore, be identical to the main analysis, so no separate exclusion was carried out. Hospital length of stay (c) was shorter on average but imprecise [(MD ≈ −3.65 days (95% CI: −7.65 to 0.36); *p* = 0.0745; I^2^ = 93.4%)], as was (d) ICU stay length [(MD ≈ −3.20 days (95% CI: −8.32 to 1.92); *p* = 0.221; I^2^ = 87.2%)]. The 95% prediction interval for hospital length of stay was very wide (−35.35 to 28.53 days), underscoring the imprecision of this estimate. The high heterogeneity, with I^2^ values of 93.4% and 87.2%, respectively, observed for these duration-based outcomes, indicates that differences in patient severity, care pathways, intervention intensity, and discharge practices likely influenced the results. Overall, the quasi-experimental evidence supports a beneficial effect of pharmacist-led care on harder clinical endpoints such as mortality and readmission, whereas its effect on duration of hospitalization remains less certain.

#### 3.5.2. Humanistic Outcomes

##### Antimicrobial Utilization (LOT/DOD and DDD)

**Figure 13:**
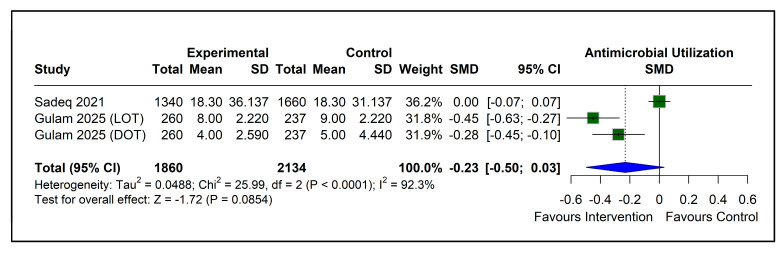
Forest plot of antimicrobial utilization with pooled analyses [31,36].

Three comparisons were made with 3994 participants. Overall, antimicrobial use tended to be lower in the intervention group, but the difference was not statistically significant [(SMD = −0.23 (95% CI: −0.50 to 0.03); *p* = 0.0854)], as shown in Figure 13. There was considerable variability between studies (I^2^ = 92.3%). Gulam (2025) showed clear reductions, but Sadeq (2021) did not. Leave-one-out sensitivity did not alter the non-significant conclusion. The 95% prediction interval was very wide (SMD: −3.88 to 3.42), consistent with the considerable variability between studies. The high variability across studies likely reflects differences in how antimicrobial use was measured.

#### 3.5.3. Economic Outcomes

##### Healthcare Resource Utilization/Clinic Visits

**Figure 14:**
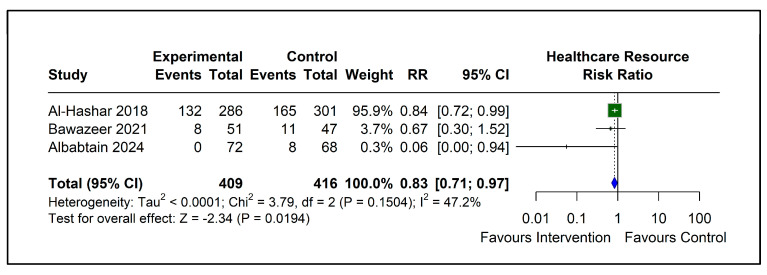
Forest plot of risk ratios for healthcare resource utilization [22,24,28].

Three studies (825 participants) were pooled for unplanned healthcare use, as described in Figure 14. The intervention significantly reduced risk [(RR = 0.83 (95% CI: 0.71 to 0.97); *p* = 0.0194)]. The 95% prediction interval was wide and crossed the null (RR: 0.21 to 3.34). The heterogeneity was moderate (I^2^ = 47.2%; τ^2^ < 0.0001; Q = 3.79; *p* = 0.150). Leave-one-out analysis indicated the pooled estimate was driven largely by Al Hashar (2018). Overall, the pooled estimate favored a reduction in healthcare resource utilization where pharmacist-led interventions were employed, although the wide prediction interval and the small number of studies indicate this effect may not be consistent across settings.

### 3.6. Non-Poolable Outcomes (Narrative Synthesis)

Non-poolable outcomes were heterogeneous but broadly favorable. Clinically, interventions significantly reduced BMI (*p* < 0.005) and 10-year coronary heart disease risk (*p* < 0.001), while increasing target attainment for BP and HbA1c (*p* = 0.0213) [20]. Gestational interventions successfully reduced pre-eclampsia (*p* = 0.014) and cesarean rates (7.1% vs. 18.2%; *p* = 0.028) [19]. However, outcomes for hypoglycemia, renal function, and anticoagulation control were mixed or non-significant (*p* = 0.65) [29]. Humanistic measures demonstrated improvements in self-reported adherence (*p* = 0.024) [30], lifestyle scores (*p* < 0.01), and patient satisfaction (*p* = 0.00001), which increased significantly [33]. Lifestyle and knowledge scores of patients increased in various studies (*p* < 0.01–*p* < 0.001) [17,20,25,26]. Process and safety gains were notable as pharmacist prescribers and antimicrobial stewardship programs significantly reduced medication errors, and antibiotic de-escalation following stewardship interventions improved (62.0% vs. 40.6%; *p* < 0.001) [35]. Meanwhile, preventable ADEs were significantly associated with non-adherence (*p* = 0.049) and medication discrepancies (*p* = 0.037) [22,34,35]. Economically, interventions sometimes increased outpatient clinic attendance (*p* < 0.005) [18,23]. In stewardship settings, outpatient or clinic attendance sometimes increased [31,35], though one stewardship phase raised antimicrobial costs due to linezolid (*p* = 0.678) and, when excluded, revealed a 37% cost reduction (*p* = 0.008) [32]. Overall, while the magnitude of benefit varied, pharmacist-led care was associated with improvements in process and humanistic metrics alongside targeted clinical advantages. Detailed statistical findings for non-poolable outcomes can be found in Supplementary Table S5.

## 4. Discussion

Across randomized and quasi-experimental evidence, pharmacist-led interventions were associated with clinically meaningful but outcome-specific benefits [53]. In RCTs, pharmacist-delivered care significantly improved HbA1c, fasting glucose, LDL-C, DBP, medication adherence, medication knowledge, and several SF-36 domains, consistent with prior systematic reviews [54,55,56]. The effects on SBP, total cholesterol, triglycerides, HDL-C, and pooled adherence/knowledge outcomes were favorable but statistically uncertain, reflecting well-documented heterogeneity in pharmacist-led programs [57,58]. Quasi-experimental evidence showed statistically consistent reductions in mortality and readmissions (I^2^ = 0%), while hospital and ICU length of stay remained inconsistent, patterns also noted in broader antimicrobial stewardship (AMS) evaluations [59,60]. Variability has been reported in inpatient pharmacy and AMS evaluations [31,61]. It should be emphasized that an I^2^ of 0% reflects agreement between study estimates rather than freedom from bias. Non-randomized studies that share the same design-related biases may produce homogeneous yet similarly biased results [45,46,47]. The mortality and readmission findings derive from pre–post and historical–control designs that are vulnerable to secular trends, regression to the mean, and co-interventions occurring alongside the pharmacist-led service, and none of the included studies fully adjusted for these. Accordingly, these outcomes were rated as low to very low certainty under GRADE (Table 3) on the basis of risk of bias rather than inconsistency and are best regarded as promising signals requiring confirmation in controlled designs [62]. Humanistic outcomes showed a split pattern, pooled RCT effects for adherence were borderline significant, and knowledge was statistically significant [63], whereas all SF-36 quality-of-life domains improved significantly, supporting evidence that pharmacist-led education and follow-up enhance patient-reported outcomes [64].

I report significant variability (I^2^ = 99%) for HbA1c with this meta-analysis, which is not uncommon with complex health services interventions. It is likely that this is due to the population, intervention intensity, design and outcomes, rather than chance [65]. Other meta-analyses of pharmacist services report variability, like Coutureau et al., who found significant variance in HbA1c (12 studies), including both RCTs and quasi-experimental designs, and conducted subgroup-analyses to explore variability [55]. Systematic reviews of complex interventions commonly find heterogeneity of effect sizes that cannot be fully explained, indicating that the interventions operate within systems where genuine variability in effect size is a part of system function rather than a hindrance [66]. Another recent systematic review from the Middle East found significant changes in HbA1c from pharmacist intervention, similar to our results [67].

The findings of significant HbA1c and LDL-C changes with RCTs agree with systematic reviews that report improvement in HbA1c and other cardiovascular risk factors [54,57]. Alabkal et al. reported reduced HbA1c and SBP, but varied effects on adherence and quality of life [54]. Similarly, Assaf et al. reported improved glycemic control and, occasionally, adherence [57]. We found non-significant SBP and significant DBP effects, with high variance, consistent with reports that pharmacist interventions reduce BP, but the effect size varies [68,69]. Variability is likely attributable to differences in intervention intensity, including whether medication optimization accompanies patient education. High variance (>95%) is common in in-service interventions; the characteristics of controls, patients under treatment and protocols being followed contribute to this effect [65]. This is shown by the broad CI for HbA1c and should be taken into account when considering the overall effect size, and the significance and direction of the overall effect did not change in the leave-one-out analyses.

For lipid outcomes, the significant LDL-C reduction in the present synthesis aligns with the expectation that pharmacist interventions can improve atherogenic lipid fractions through medication optimization and adherence [54,69]. However, the absence of statistically significant changes in total cholesterol, triglycerides, and HDL-C, despite a favorable direction, is consistent with prior meta-analyses reporting mixed lipid outcomes [54]. These inconsistencies likely reflect differences in baseline lipid levels, study populations, and intervention intensity, as well as methodological variability.

In AMS, the findings demonstrate insignificant but directionally favorable effects. While pooled analyses suggested reductions in antibiotic utilization, these were not consistently significant and showed high heterogeneity [31,61]. AMS interventions can reduce antibiotic days of therapy, while length of stay (LOS) effects are often non-significant, with some studies showing reduced readmissions and mortality, especially when pharmacists join multidisciplinary stewardship teams [31]. The broader AMS literature highlights variability in effectiveness depending on implementation strategies and contextual factors [68]. Quasi-experimental studies reinforce this heterogeneity. Difference-in-differences analyses have shown reductions in antibiotic consumption and costs, though LOS effects vary across settings [70,71]. Audit-and-feedback interventions reduce antibiotic use and therapy duration without compromising clinical outcomes, yet their effects on mortality and readmissions remain inconsistent [71]. These findings suggest that AMS outcomes are highly context-dependent and influenced by local implementation structures.

Humanistic outcomes remain challenging to interpret. The borderline pooled adherence effect and significant knowledge effect are consistent with evidence that adherence improvements are often modest and difficult to detect, particularly when measurement approaches differ [57,72]. Systematic reviews report improved medication knowledge without consistent adherence gains, and community pharmacist-led interventions show similar variability [72]. In contrast, improvements in quality of life are more consistent. Evidence from chronic disease populations indicates that pharmacist-led care, particularly when involving medication review and follow-up, can improve patient-reported outcomes [64,73].

Data on toxicity and treatment-emergent harm warrant comment. The included studies were designed primarily to evaluate effectiveness rather than to capture toxicity systematically, so safety outcomes were reported inconsistently. Where reported, the signals were favorable: preventable adverse drug events were reduced and were significantly associated with non-adherence and medication discrepancies in the transitions-of-care setting [34], and medication errors decreased under pharmacist independent prescribing in critical care [41]. For glycemic interventions, hypoglycemia was the most relevant treatment-emergent harm and was mixed or non-significant [28]. Within antimicrobial stewardship, the interventions did not increase mortality, and antibiotic de-escalation improved, indicating no safety signal arising from reduced antibiotic exposure [37,39,41]. Overall, systematic toxicity and adverse-event data were sparse across the evidence base, which limits any firm conclusion about the safety profile of pharmacist-led interventions in the region and points to standardized harm reporting as a priority for future GCC studies.

Clinically, pharmacist-led interventions appear most effective for improving glycemic control and LDL-C, with less consistent effects on BP and other lipid parameters [54,69,74]. Effectiveness depends on intervention design, particularly the inclusion of medication therapy management and structured follow-up rather than education alone.

In inpatient and AMS settings, reductions in mortality and readmissions, along with directionally favorable results in antibiotic utilization, support the integration of pharmacists within multidisciplinary teams [31,70,71]. However, outcomes such as LOS remain context-dependent, emphasizing the need to align intervention design with local infrastructure, staffing, and measurement strategies [68,75]. Structural factors, including stewardship tools and system-level disruptions, may further influence outcomes [75].

Telehealth and tele-stewardship models offer scalable alternatives, particularly in resource-limited settings, although comparative evidence remains limited [76,77]. Given the multidimensional effects observed, evaluation frameworks should incorporate clinical, humanistic, safety, and economic outcomes to support a comprehensive assessment of pharmacist-led services [61].

### 4.1. Strengths of This Study

This study provides a comprehensive synthesis of pharmacist-led interventions across clinical, humanistic, and economic domains within GCC country settings, incorporating evidence from both randomized and quasi-experimental designs. This approach addresses an important gap in the literature, where evidence from GCC countries’ healthcare systems remains limited [61]. The use of random-effects models and sensitivity analyses enhances robustness in the presence of heterogeneity [29,31,68]. To my knowledge, evidence over the past two decades remains limited, particularly for studies integrating clinical, humanistic, and antimicrobial stewardship outcomes in GCC country settings.

### 4.2. Limitations

Interpretation is limited by substantial heterogeneity across outcomes, reflecting variations in intervention content, duration, settings, and measurement approaches, particularly for adherence and antimicrobial utilization [54,57,72,78]. Quasi-experimental findings remain susceptible to residual confounding and concurrent system-level influences [64,68,75]. Humanistic outcomes were constrained by inconsistent measurement and definitions, while economic results showed uncertainty and sensitivity to individual studies, limiting generalizability without standardized costing methods and stronger causal evidence [57,69,78]. The evidence base was also geographically uneven. Of the 20 included studies, nine originated from Saudi Arabia, seven from the United Arab Emirates, two from Qatar, one from Oman, and one from Kuwait, while no eligible study was identified from Bahrain. The pooled estimates, therefore, predominantly reflect Saudi and Emirati practice, and the findings should not be assumed to generalize uniformly across the wider GCC region. In addition, most pooled outcomes drew on only three to six studies. With fewer than ten studies, the funnel plot asymmetry and Egger regression were underpowered and were treated as exploratory [8,50]. Subgroup analyses could not reliably attribute heterogeneity, and formal meta-regression on study-level covariates was not feasible. These constraints, together with the residual confounding inherent in the non-randomized studies, are reflected in the GRADE certainty ratings and temper the strength of the conclusions [48].

## 5. Conclusions

This meta-analysis shows that pharmacist-led interventions in GCC country settings were associated with improvements in glycemic control, LDL-C, DBP, borderline medication adherence, medication knowledge, and patient-reported quality of life. Improvements in SBP, other lipid fractions, unplanned healthcare use, and antimicrobial utilization were encouraging but not statistically consistent, reflecting variation in how interventions were delivered and measured. Quasi-experimental studies demonstrated statistically consistent reductions in mortality and readmissions, while effects on hospital and ICU stay varied widely. Together, these findings highlight the growing value of pharmacist-led care in the region and the need for more standardized outcomes and clearer intervention models to guide future practice. These conclusions should be read in light of the GRADE certainty ratings, which were low to very low for most outcomes owing to substantial heterogeneity and risk of bias, and of the geographic concentration of the evidence in Saudi Arabia and the United Arab Emirates, which limits generalizability across the wider GCC region. The reductions in mortality and readmissions, derived from non-randomized designs, require confirmation in controlled studies.

## Figures and Tables

**Table 1:** Baseline characteristics of randomized studies.

S. No.	Author (Year)	Country	Study Design	Setting	Population	Sample Size (I/C)	Pharmacist Intervention	Comparator	Outcomes Measured
1	Sadik et al., 2005 [17]	UAE	Randomized controlled Trial	Hospital and outpatient clinics	Heart failure	208 (104/104)	Pharmaceutical care program with monitoring and education	Usual care	Exercise tolerance, QoL, adherence, and hospitalization
2	Al-Saffar et al., 2005 [18]	Kuwait	Open RCT (3 arms)	Psychiatric hospital outpatient clinic	Depression patients	185 (98/87)	Patient information leaflet ± pharmacist counseling	Usual care	Medication adherence and clinic attendance
3	Elnour et al., 2008 [19]	UAE	Randomized controlled trial	Hospital outpatient clinic	Gestational diabetes	165 (99/66)	Pharmaceutical care including education and self-monitoring	Usual care	HbA1c, plasma glucose, and QoL
4	Al Mazroui et al., 2009 [20]	UAE	Randomized controlled trial	Military hospital outpatient clinic	Type 2 diabetes	234 (117/117)	Pharmaceutical care program with education and adherence support	Usual care	HbA1c, BP, HRQoL, and adherence
5	El Hajj et al., 2017 [21]	Qatar	Randomized controlled trial	Ambulatory pharmacies	Adult smokers	314 (167/147)	Structured pharmacist smoking cessation program	Usual care	Smoking cessation rate
6	Al-Hashar et al., 2018 [22]	Oman	Randomized controlled trial	Tertiary hospital	Hospitalized medical patients	587 (286/301)	Medication reconciliation; discharge counselling	Usual care	Preventable ADEs and healthcare resource use
7	Tourkmani et al., 2018 [23]	Saudi Arabia	Randomized controlled trial	Diabetes clinic	Type 2 diabetes	263 (195/68)	Multidisciplinary integrated care	Usual care	HbA1c, fasting glucose, lipid profile, and BP
8	Bawazeer et al., 2021 [24]	Saudi Arabia	Randomized controlled trial	Teaching hospital	Patients on high-risk medications	98 (51/47)	Pharmacist-led medication reconciliation and counselling	Usual care	30-day readmission, healthcare use, and satisfaction
9	Khan et al., 2022 [25]	Saudi Arabia	Pre–post, randomized	Community pharmacy	Type 2 diabetes	109 (54/55)	Pharmacist-led diabetes education and telepharmacy follow-up	Usual care	HbA1c, adherence, knowledge, and lipid profile
10	Ibrahim et al., 2022 [26]	UAE	2-arm randomized clinical trial	Community pharmacies	Hypertension	239 (119/120)	Telepharmacy services with pharmacist monitoring	Usual care	SBP, DBP, adherence, knowledge, and DRPs
11	El-Deyarbi et al., 2024 [27]	UAE	Randomized controlled trial	Ambulatory diagnostic center	Type 2 diabetes	192 (94/98)	Medication therapy management, counselling, and medication booklet	Usual care	Medication adherence and drug-related problems
12	Albabtain et al., 2024 [28]	Saudi Arabia	Randomized controlled trial	Community pharmacy	Uncontrolled diabetes	160 (80/80)	MTM program	Usual care	HbA1c, BP, hospitalization, and adherence
13	Mekdad et al., 2025 [29]	Saudi Arabia	Prospective randomized study	Cardiac center hospital	Diabetes with cardiac conditions	1000 (500/500)	Clinical pharmacist management in multidisciplinary care	Usual care	HbA1c, fasting glucose, and hypoglycemia

Abbreviations: I/C: intervention/control.

**Table 2:** Baseline characteristics of quasi-experimental studies.

S. No.	Author (Year)	Country	Study Design	Setting	Population	Sample Size (I/C)	Pharmacist Intervention	Comparator	Outcomes Measured
1	Alajmi et al., 2017 [30]	Saudi Arabia	Prospective non-randomized controlled interventional study	Neurology outpatient clinic	Adult epilepsy patients	60 (30/30)	Pharmacist-led educational interview and adherence counselling	Standard follow-up without intervention	Medication adherence (Morisky score)
2	Sadeq et al., 2021 [31]	UAE	Controlled non-randomized intervention study	Hospital wards and ICU	Hospitalized adult patients receiving antibiotics	3000 (1340/1660)	Multidisciplinary antimicrobial stewardship program with pharmacist-led case review	Usual care stewardship practices	LOS, readmission, mortality, and antibiotic utilization
3	Haseeb et al., 2021 [32]	Saudi Arabia	Quasi-experimental pre–post study	Intensive care unit	ICU patients receiving antimicrobials	135/169 (pre vs. post phases)	Multidisciplinary antimicrobial stewardship program with pharmacist audit and feedback	Pre-intervention phase	Antibiotic consumption (DDD/100 bed-days) and cost
4	El Hajj et al., 2023 [33]	Qatar	Prospective quasi-experimental (natural allocation)	Cardiology hospital	Patients discharged after acute coronary syndrome	373 (111 intervention/ 120 usual care/ 142 control)	Pharmacist-led discharge medication reconciliation, counselling, and follow-up sessions	Usual care or weekend discharge control	All-cause hospitalization and cardiac-related readmission
5	Sarkhi et al., 2024 [34]	Saudi Arabia	Quasi-experimental (pre–post with comparator phase)	Tertiary hospital	Hospitalized patients receiving restricted antibiotics	167/190 (pre vs. post phase)	Multidisciplinary antimicrobial stewardship program with prospective audit and feedback by clinical pharmacists	Pre-intervention phase (usual care)	Antibiotic utilization (DDD and DOT), clinical cure, LOS, ICU stay, mortality, and readmission
6	Ibrahim et al., 2025 [35]	Saudi Arabia	Prospective controlled interventional study	Intensive care unit (ICU)	Critically ill patients	40 (20/20)	Pharmacist independent prescribing and medication management	Physician-based prescribing	Medication errors, LOS, mortality, cost-effectiveness, and guideline adherence
7	Gulam et al., 2025 [36]	UAE	Quasi-experimental (pre–post with historical control)	Tertiary hospital	Hospitalized patients receiving targeted antimicrobials	497 (260/237)	Pharmacist-led prospective audit and feedback antimicrobial stewardship intervention	Historical control (pre-implementation period)	LOS, length of therapy, antibiotic utilization (DDD and DOT), readmission, and mortality

Abbreviations: I/C: intervention/control.

**Table 3:** GRADE summary of findings. Certainty was rated per outcome and downgraded for risk of bias, inconsistency, and imprecision. Randomized evidence began at high certainty and non-randomized evidence at low certainty.

Outcome	Studies (Design)	Pooled Effect (95% CI)	95% Prediction Interval	I^2^	Reasons for Downgrading	Certainty
HbA1c (primary)	6 RCT	MD: −1.13% (−2.21 to −0.05)	−4.50 to 2.23%	99%	Inconsistency (very serious); imprecision (CI near null)	Very low
Fasting blood glucose	4 RCT	MD: −1.10 mmol/L (−1.93 to −0.28)	−5.46 to 3.21 mmol/L	>95%	Inconsistency (very serious); small-study effects	Very low
LDL cholesterol	4 RCT	MD: −0.30 mmol/L (−0.53 to −0.07)	−1.35 to 0.75 mmol/L	77–82%	Inconsistency (serious); imprecision	Low
Diastolic BP	6 RCT	MD: −4.11 mmHg (−6.83 to −1.39)	−13.81 to 5.59 mmHg	86%	Inconsistency (serious)	Low
Systolic BP	6 RCT	Non-significant (*p* = 0.108)	−13.90 to 6.43 mmHg	73%	Inconsistency; imprecision (CI crosses null)	Low
Medication knowledge	3 RCT	Favorable (*p* = 0.015)	Not estimable (k = 3)	89%	Inconsistency (serious); imprecision	Low
Medication adherence	4 RCT	Borderline (*p* = 0.050)	RR: 0.29 to 9.59	93%	Inconsistency (very serious); imprecision	Very low
Mortality	6 non-randomized	RR: 0.76 (0.66 to 0.87)	RR: 0.54 to 1.07	0%	Risk of bias (serious, ROBINS-I); confounding	Low to very low
Readmissions	3 non-randomized	RR: 0.72 (0.64 to 0.82)	RR: 0.32 to 1.65	0%	Risk of bias (serious, ROBINS-I); confounding	Low to very low
Hospital length of stay	3 non-randomized	MD: −3.65 (−7.65 to 0.36)	−35.35 to 28.53	93%	Risk of bias, inconsistency, and imprecision	Very low
Antimicrobial utilization	3 comparisons	SMD: −0.23 (−0.50 to 0.03)	SMD: −3.88 to 3.42	92%	Inconsistency (very serious); imprecision	Very low
Unplanned healthcare use	3 studies	RR: 0.83 (0.71 to 0.97)	RR: 0.21 to 3.34	47%	Risk of bias; imprecision (few studies)	Low

Abbreviations: BP, blood pressure. CI, confidence interval. LDL, low-density lipoprotein. MD, mean difference. RCT, randomized controlled trial. RR, risk ratio. SMD, standardized mean difference.

## Data Availability

No new data were created or analyzed in this study. Data sharing is not applicable to this article.

## Supplementary Materials

The following supporting information can be downloaded at https://www.mdpi.com/article/10.3390/pharmacy14040102/s1.

## References

[1] Al-Jedai A., Qaisi S., Al-Meman A. (2016). Pharmacy Practice and the Health Care System in Saudi Arabia. Can. J. Hosp. Pharm..

[2] Alameri H.F., McMahon G.T., Al Jenaibi F.H., Husseiny A.M. (2023). Abu Dhabi’s Journey Towards Excellence in Continuing Medical Education. J. CME.

[3] Al Busaidi N., Alweqayyan A., Al Zaabi A., Mahboub B., Al-Huraish F., Hameed M., Al-Ahmad M., Khadadah M., Al Lawati N., Behbehani N. (2022). Gulf Asthma Diagnosis and Management in Adults: Expert Review and Recommendations. Open Respir. Med. J..

[4] Al Rahbi H.A., Al-Sabri R.M., Chitme H.R. (2014). Interventions by pharmacists in out-patient pharmaceutical care. Saudi Pharm. J..

[5] Jaam M., Naseralallah L.M., Hussain T.A., Pawluk S.A. (2021). Pharmacist-led educational interventions provided to healthcare providers to reduce medication errors: A systematic review and meta-analysis. PLoS ONE.

[6] Alsultan M.S., Khurshid F., Mayet A.Y., Al-Jedai A.H. (2012). Hospital pharmacy practice in Saudi Arabia: Dispensing and administration in the Riyadh region. Saudi Pharm. J..

[7] Al-Ruthia Y.S., Balkhi B., AlGhadeer S., Mansy W., AlSanawi H., AlGasem R., AlMutairi L., Sales I. (2017). Relationship between health literacy and body mass index among Arab women with polycystic ovary syndrome. Saudi Pharm. J..

[8] Higgins J.P.T., Thomas J., Chandler J., Cumpston M., Li T., Page M.J. (2024). Cochrane Handbook for Systematic Reviews of Interventions.

[9] Page M.J., Moher D., Bossuyt P.M., Boutron I., Hoffmann T.C., Mulrow C.D., Shamseer L., Tetzlaff J.M., Akl E.A., Brennan S.E. (2021). PRISMA 2020 explanation and elaboration: Updated guidance and exemplars for reporting systematic reviews. BMJ.

[10] Moher D., Liberati A., Tetzlaff J., Altman D.G. (2009). Preferred reporting items for systematic reviews and meta-analyses: The PRISMA statement. BMJ.

[11] Amir-Behghadami M., Janati A. (2020). Population, Intervention, Comparison, Outcomes and Study (PICOS) design as a framework to formulate eligibility criteria in systematic reviews. Emerg. Med. J..

[12] Wong L.H., Tay E., Heng S.T., Guo H., Kwa A.L.H., Ng T.M., Chung S.J., Somani J., Lye D.C., Chow A. (2021). Hospital Pharmacists and Antimicrobial Stewardship: A Qualitative Analysis. Antibiotics.

[13] Wu J.H.C., Khalid F., Langford B.J., Beahm N.P., McIntyre M., Schwartz K.L., Garber G., Leung V. (2021). Community Pharmacist Prescribing of Antimicrobials: A Systematic Review From an Antimicrobial Stewardship Perspective. Can. Pharm. J..

[14] Fares R., Marjorie B., Chaballe C., Crunenberg R. (2025). The outcomes of pharmacist-led pharmaceutical care within community pharmacies: An overview of systematic reviews. Res. Soc. Soc. Adm. Pharm..

[15] Smith-Ray R., Feng L., Singh T., Rudkin K., Emmons S., Groves E., Kirkham H. (2024). Pharmacists as clinical care partners: How a pharmacist-led intervention is associated with improved medication adherence in older adults with common chronic conditions. J. Manag. Care Spec. Pharm..

[16] Microsoft C. (2024). Microsoft Excel, Version 365 (Office 365).

[17] Sadik A., Yousif M., McElnay J. (2005). Pharmaceutical care of patients with heart failure. Br. J. Clin. Pharmacol..

[18] Al-Saffar N., Deshmukh A.A., Carter P., Adib S.M. (2005). Effect of information leaflets and counselling on antidepressant adherence: Open randomised controlled trial in a psychiatric hospital in Kuwait. Int. J. Pharm. Pract..

[19] Elnour A., El Mugammar I., Jaber T., Revel T., McElnay J.C. (2008). Pharmaceutical care of patients with gestational diabetes mellitus. J. Eval. Clin. Pract..

[20] Al Mazroui N.R., Kamal M.M., Ghabash N.M., Yacout T.A., Kole P.L., McElnay J.C. (2009). Influence of pharmaceutical care on health outcomes in patients with Type 2 diabetes mellitus. Br. J. Clin. Pharmacol..

[21] El Hajj M.S., Kheir N., Al Mulla A.M., Shami R., Fanous N., Mahfoud Z.R. (2017). Effectiveness of a pharmacist-delivered smoking cessation program in the State of Qatar: A randomized controlled trial. BMC Public Health.

[22] Al-Hashar A., Al-Zakwani I., Eriksson T., Sarakbi A., Al-Zadjali B., Al Mubaihsi S., Al Za’abi M. (2018). Impact of medication reconciliation and review and counselling, on adverse drug events and healthcare resource use. Int. J. Clin. Pharm..

[23] Tourkmani A.M., Abdelhay O., Alkhashan H.I., Alaboud A.F., Bakhit A., Elsaid T., Alawad A., Alobaikan A., Alqahtani H., Alqahtani A. (2018). Impact of an integrated care program on glycemic control and cardiovascular risk factors in patients with type 2 diabetes in Saudi Arabia: An interventional parallel-group controlled study. BMC Fam. Pract..

[24] Bawazeer G., Sales I., Alsunaidi A., Aljahili S., Aljawadi M.H., Almalag H.M., Alkofide H., Adam Mahmoud M., Alayoubi F., Aljohani M. (2021). Student-Led discharge counseling program for High-Risk medications in a teaching hospital in Saudi Arabia: A pilot study. Saudi Pharm. J..

[25] Khan Y.H., Alzarea A.I., Alotaibi N.H., Alatawi A.D., Khokhar A., Alanazi A.S., Butt M.H., Alshehri A.A., Alshehri S., Alatawi Y. (2022). Evaluation of Impact of a Pharmacist-Led Educational Campaign on Disease Knowledge, Practices and Medication Adherence for Type-2 Diabetic Patients: A Prospective Pre- and Post-Analysis. Int. J. Environ. Res. Public Health.

[26] Ibrahim O.M., Al Meslamani A.Z., Ibrahim R., Kaloush R., Al Mazrouei N. (2022). The impact of telepharmacy on hypertension management in the United Arab Emirates. Pharm. Pract..

[27] El-Deyarbi M., Ahmed L., King J., Abubackar S., Al Juboori A., Mansour N.A., Aburuz S. (2024). The effects of multifactorial pharmacist-led intervention protocol on medication optimisation and adherence among patients with type 2 diabetes: A randomised control trial. F1000Research.

[28] Albabtain B., Bawazeer G., Paudyal V., Cheema E., Alqahtani A., Bahatheq A., Price M.J., Hadi M.A. (2024). Impact of a community pharmacy-based medication therapy management program on clinical and humanistic outcomes in patients with uncontrolled diabetes: A randomised controlled trial. Sci. Rep..

[29] Mekdad S., Alsayed L., Alkhulaif S. (2025). The role of clinical pharmacists in improving diabetic care of hospitalized heart patients. Diabetes Epidemiol. Manag..

[30] AlAjmi R., Al-Aqeel S., Baz S. (2017). The impact of a pharmacist-led educational interview on medication adherence of Saudi patients with epilepsy. Patient Prefer. Adherence.

[31] Sadeq A.A., Shamseddine J.M., Babiker Z.O.E., Nsutebu E.F., Moukarzel M.B., Conway B.R., Hasan S.S., Conlon-Bingham G.M., Aldeyab M.A. (2021). Impact of multidisciplinary team escalating approach on antibiotic stewardship in the United Arab Emirates. Antibiotics.

[32] Haseeb A., Faidah H.S., Al-Gethamy M., Iqbal M.S., Barnawi A.M., Elahe S.S., Bukhari D.N., Noor Al-Sulaimani T.M., Fadaaq M., Alghamdi S. (2021). Evaluation of a multidisciplinary antimicrobial stewardship program in a Saudi critical care unit: A quasi-experimental study. Front. Pharmacol..

[33] El Hajj M.S., Kaddoura R., Abu Yousef S.E., Orabi B., Awaisu A., AlYafei S., Shami R., Mahfoud Z.R. (2023). Effectiveness of a structured pharmacist-delivered intervention for patients post-acute coronary syndromes on all-cause hospitalizations and cardiac-related hospital readmissions: A prospective quasi-experimental study. Int. J. Clin. Pharm..

[34] Sarkhi K.A., Eljaaly K., Kaki R., Bahamdan R., Alghamdi S.A., Baharith M.O., Thabit A.K. (2024). Impact of a multidisciplinary antimicrobial stewardship program on antibiotic utilization and clinical outcomes at a tertiary hospital in Saudi Arabia: A quasi-experimental study. Expert Rev. Anti-Infect. Ther..

[35] Ibrahim S.E., Al-Gadi S., El-Sisi M.A., Alrifai M.A., Maher M., Badawy A.I., Saadat H.M., Abdoh H.Y., Hegazy M.M., Abdalhamid R.S. (2025). Impact of pharmacist independent prescription on critical care: A prospective interventional study in Madinah, Saudi Arabia. Trop. J. Pharm. Res..

[36] Gulam S.M., Thomas D., Ahamed F., Baker D.E. (2025). Prospective Audit and Feedback of Targeted Antimicrobials Use at a Tertiary Care Hospital in the United Arab Emirates. Antibiotics.

[37] Core T. (2023). R: A Language and Environment for Statistical Computing.

[38] Posit T. (2012). RStudio: Integrated Development Environment for R.

[39] Microsoft Corporation (2026). Copilot (April 2026 Version).

[40] Google Gemini, Alphabet Inc (2026). Google AI Tools.

[41] Borenstein M., Higgins J.P., Hedges L.V., Rothstein H.R. (2017). Basics of meta-analysis: I2 is not an absolute measure of heterogeneity. Res. Synth. Methods.

[42] Rücker G., Schwarzer G., Carpenter J.R., Schumacher M. (2008). Undue reliance on I 2 in assessing heterogeneity may mislead. BMC Med. Res. Methodol..

[43] Higgins J.P., Thompson S.G., Deeks J.J., Altman D.G. (2003). Measuring inconsistency in meta-analyses. BMJ.

[44] IntHout J., Ioannidis J.P., Rovers M.M., Goeman J.J. (2016). Plea for routinely presenting prediction intervals in meta-analysis. BMJ Open.

[45] Guyatt G.H., Oxman A.D., Kunz R., Woodcock J., Brozek J., Helfand M., Alonso-Coello P., Glasziou P., Jaeschke R., Akl E.A. (2011). GRADE guidelines: 7. Rating the quality of evidence—Inconsistency. J. Clin. Epidemiol..

[46] Guyatt G.H., Oxman A.D., Kunz R., Brozek J., Alonso-Coello P., Rind D., Devereaux P., Montori V.M., Freyschuss B., Vist G. (2011). GRADE guidelines 6. Rating the quality of evidence—Imprecision. J. Clin. Epidemiol..

[47] Guyatt G.H., Oxman A.D., Vist G., Kunz R., Brozek J., Alonso-Coello P., Montori V., Akl E.A., Djulbegovic B., Falck-Ytter Y. (2011). GRADE guidelines: 4. Rating the quality of evidence—Study limitations (risk of bias). J. Clin. Epidemiol..

[48] Schünemann H.J., Cuello C., Akl E.A., Mustafa R.A., Meerpohl J.J., Thayer K., Morgan R.L., Gartlehner G., Kunz R., Katikireddi S.V. (2019). GRADE guidelines: 18. How ROBINS-I and other tools to assess risk of bias in nonrandomized studies should be used to rate the certainty of a body of evidence. J. Clin. Epidemiol..

[49] Guyatt G.H., Oxman A.D., Vist G.E., Kunz R., Falck-Ytter Y., Alonso-Coello P., Schünemann H.J. (2008). GRADE: An emerging consensus on rating quality of evidence and strength of recommendations. BMJ.

[50] Sterne J.A.C., Sutton A.J., Ioannidis J.P.A., Terrin N., Jones D.R., Lau J., Carpenter J., Rücker G., Harbord R.M., Schmid C.H. (2011). Recommendations for examining and interpreting funnel plot asymmetry in meta-analyses of randomised controlled trials. BMJ.

[51] Sterne J.A., Savović J., Page M.J., Elbers R.G., Blencowe N.S., Boutron I., Cates C.J., Cheng H.-Y., Corbett M.S., Eldridge S.M. (2019). RoB 2: A revised tool for assessing risk of bias in randomised trials. BMJ.

[52] Sterne J.A., Hernán M.A., Reeves B.C., Savović J., Berkman N.D., Viswanathan M., Henry D., Altman D.G., Ansari M.T., Boutron I. (2016). ROBINS-I: A tool for assessing risk of bias in non-randomised studies of interventions. BMJ.

[53] Khobrani M., Alshahrani S.M. (2025). Clinical pharmacist-led interventions and their impact on outcomes in patients with bipolar I disorder: A systematic review and meta-analysis. Front. Med..

[54] Alabkal R.M., Medlinskiene K., Silcock J., Graham A. (2023). Impact of pharmacist-led interventions to improve clinical outcomes for adults with type 2 diabetes at risk of developing cardiovascular disease: A systematic review and meta-analysis. J. Pharm. Pract..

[55] Coutureau C., Slimano F., Mongaret C., Kanagaratnam L. (2022). Impact of pharmacists-led interventions in primary care for adults with type 2 diabetes on HbA1c levels: A systematic review and meta-analysis. Int. J. Environ. Res. Public Health.

[56] Alqahtani S.S. (2026). Impact of Clinical Pharmacist Educational Intervention on Lipid Profile and its Clinical Outcomes. Saudi Med. J..

[57] Al Assaf S., Zelko R., Hanko B. (2022). The effect of interventions led by community pharmacists in primary care for adults with type 2 diabetes mellitus on therapeutic adherence and HbA1c levels: A systematic review. Int. J. Environ. Res. Public Health.

[58] Bennetts J., White J., Croft H., Cooper J., McIvor D., Eadie N., Appay M., Sverdlov A.L., Ngo D. (2024). Pharmacist-led medication management services: A qualitative exploration of transition-of-care cardiovascular disease patient experiences. BMJ Open.

[59] Weber C., Meyer-Massetti C., Schönenberger N. (2025). Pharmacist-led interventions at hospital discharge: A scoping review of studies demonstrating reduced readmission rates. Int. J. Clin. Pharm..

[60] Holden D.N., Groth C.M., Acquisto N.M., Eche I.M., Ferguson N., Lahey S., Buckley M. (2026). Measuring Critical Care Pharmacist Value: A Scoping Review of the Effect of Critical Care Pharmacist Activities on Clinically Relevant Outcomes. J. Am. Coll. Clin. Pharm..

[61] Elshenawy R.A., Umaru N., Alharbi A.B., Aslanpour Z. (2023). Antimicrobial stewardship implementation before and during the COVID-19 pandemic in the acute care settings: A systematic review. BMC Public Health.

[62] Murad M.H., Mustafa R.A., Schünemann H.J., Sultan S., Santesso N. (2017). Rating the certainty in evidence in the absence of a single estimate of effect. BMJ Evid.-Based Med..

[63] Deng Z.J., Gui L., Chen J., Peng S.S., Ding Y.F., Wei A.H. (2023). Clinical, economic and humanistic outcomes of medication therapy management services: A systematic review and meta-analysis. Front. Pharmacol..

[64] Christopher C.M., Kc B., Blebil A., Alex D., Ibrahim M.I.M., Ismail N., Alrasheedy A.A. (2021). Clinical and humanistic outcomes of community pharmacy-based healthcare interventions regarding medication use in older adults: A systematic review and meta-analysis. Healthcare.

[65] Choi G.J., Kang H. (2025). Heterogeneity in meta-analyses: An unavoidable challenge worth exploring. Korean J. Anesthesiol..

[66] Burton C. (2012). Heavy tailed distributions of effect sizes in systematic reviews of complex interventions. PLoS ONE.

[67] Bakhsh H.T. (2025). Impact of pharmacist interventions on health outcomes of patients with type 2 diabetes mellitus in the middle east: A systematic review. Integr. Pharm. Res. Pract..

[68] Okoro R.N., Nduaguba S.O. (2021). Community pharmacists on the frontline in the chronic disease management: The need for primary healthcare policy reforms in low and middle income countries. Explor. Res. Clin. Soc. Pharm..

[69] Costa S., Guerreiro J., Teixeira I., Helling D.K., Pereira J., Mateus C. (2022). Cost-effectiveness and cost-utility of hypertension and hyperlipidemia collaborative management between pharmacies and primary care in portugal alongside a trial compared with usual care (USFarmácia^®^). Front. Pharmacol..

[70] Wang Y., Zhou C., Liu C., Liu S., Liu X., Li X. (2022). The impact of pharmacist-led antimicrobial stewardship program on antibiotic use in a county-level tertiary general hospital in China: A retrospective study using difference-in-differences design. Front. Public Health.

[71] Xu J., Huang J., Yu Y., Zhou D., Wang Y., Xue S., Shang E., Sun J., Ding X., Shi L. (2022). The impact of a multifaceted pharmacist-led antimicrobial stewardship program on antibiotic use: Evidence from a quasi-experimental study in the department of vascular and interventional radiology in a Chinese tertiary hospital. Front. Pharmacol..

[72] Lambert M., Smit C.C., De Vos S., Benko R., Llor C., Paget W.J., Briant K., Pont L., Van Dijk L., Taxis K. (2022). A systematic literature review and meta-analysis of community pharmacist-led interventions to optimise the use of antibiotics. Br. J. Clin. Pharmacol..

[73] Schumacher P.M., Becker N., Tsuyuki R.T., Griese-Mammen N., Koshman S.L., McDonald M.A., Bouvy M., Rutten F.H., Laufs U., Böhm M. (2021). The evidence for pharmacist care in outpatients with heart failure: A systematic review and meta-analysis. ESC Heart Fail..

[74] Iqbal M.Z., Alqahtani S.S., Mubarak N., Shahid S., Mohammed R., Mustafa A., Khan A.H., Iqbal M.S. (2024). The influence of pharmacist-led collaborative care on clinical outcomes in type 2 diabetes mellitus: A multicenter randomized control trial. Front. Public Health.

[75] Roger P.-M., Lesselingue D., Gérard A., Roghi J., Quint P., Un S., Chincholle A., Assi A., Bouchard O., Javaudin V. (2024). Antibiotic consumption 2017–2022 in 30 private hospitals in France: Impact of antimicrobial stewardship tools and COVID-19 pandemic. Antibiotics.

[76] Pierce J., Stevens M.P. (2021). The emerging role of telehealth in antimicrobial stewardship: A systematic review and perspective. Curr. Treat. Options Infect. Dis..

[77] Andrzejewski C., McCreary E.K., Khadem T., Abdel-Massih R.C., Bariola J.R. (2021). Tele-antimicrobial stewardship programs: A review of the literature and the role of the pharmacist. J. Am. Coll. Clin. Pharm..

[78] Santana E.P.C., Javarini H.R.V., de Araújo D.C.S.A., Cerqueira-Santos S., Reis T.M., dos Santos-Junior G.A., Rocha K.S.S. (2025). Does drug dispensing influence patients’ medication knowledge and medication adherence? A systematic review and meta-analysis. BMC Health Serv. Res..

