# Supplementary material for: Impact of Pharmacist-Led Interventions on Patient Outcomes in Gulf Cooperation Council Countries: A Systematic Review and Meta-Analysis
Source: Pharmacy (Basel). 2026 Jul 6;14(4):102. doi: 10.3390/pharmacy14040102 (PMC13415103; doi:10.3390/pharmacy14040102)

1) Clinical outcomes
a) Randomized controlled Trials (RCTs)
i) HbA1C

(a) Sensitivity analysis for HbA1c

Omitted Study	MD	95%-CI	P-value	Tau2	Tau	I2
Omitting Elnour 2008	-1.33	[-2.57; -0.09]	0.0355	1.9697	1.4034	98.9%
Omitting Al Mazroui 2009	-1.08	[-2.40; 0.24]	0.1082	2.2333	1.4944	99.1%
Omitting Tourkmani 2018	-0.63	[-1.12; -0.13]	0.0128	0.2942	0.5424	96.4%
Omitting Khan 2022	-1.32	[-2.57; -0.07]	0.0389	2.0037	1.4155	99.1%
Omitting Albabtain 2024	-1.30	[-2.55; -0.04]	0.0435	2.0449	1.4300	99.2%
Omitting Mekdad 2025	-1.16	[-2.48; 0.16]	0.0853	2.2483	1.4994	99.2%
**Random effects model**	**-1.13**	**[-2.21; -0.05]**	**0.0396**	**1.7963**	**1.3403**	**99.0%**

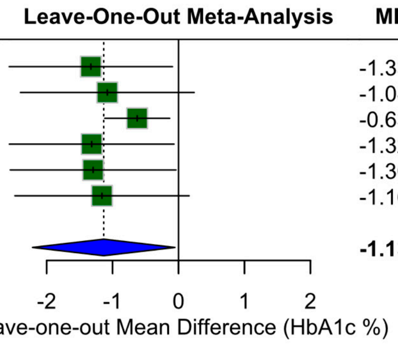

(b) Publication Bias for HbA1c

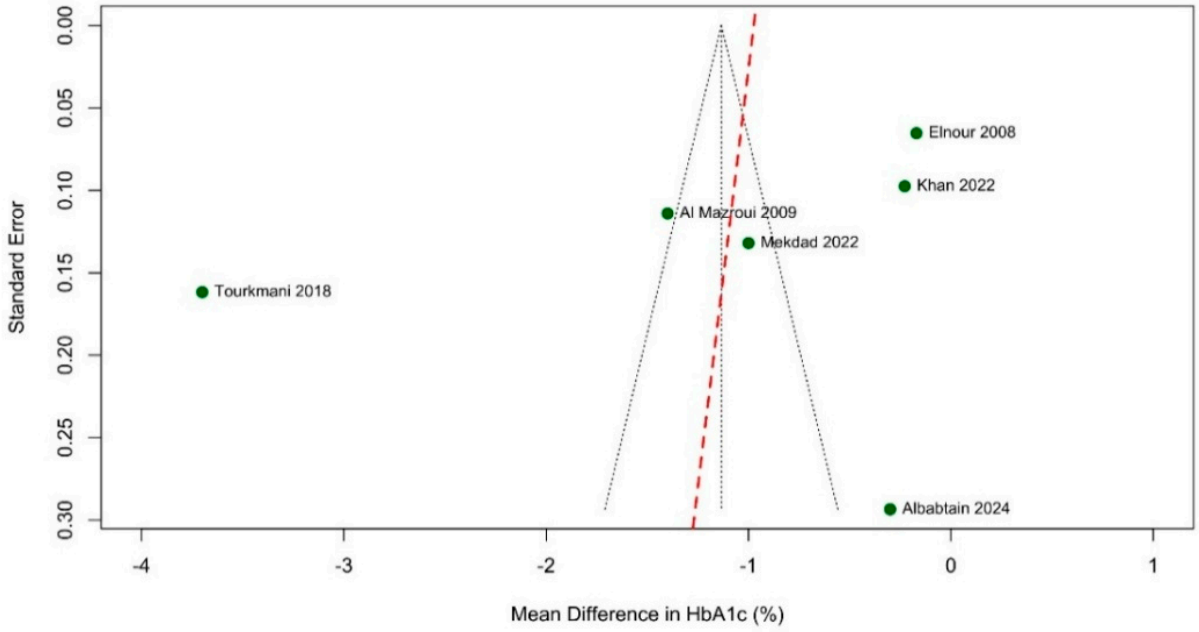

ii) Fasting Blood Glucose (FBG)

(a) Sensitivity analysis for FBG

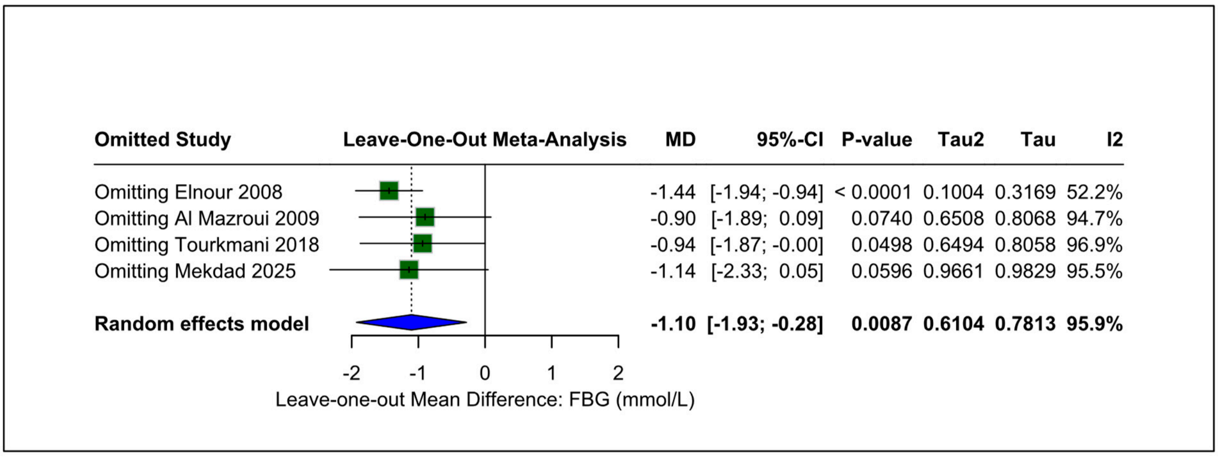

(b) Publication Bias for FBG

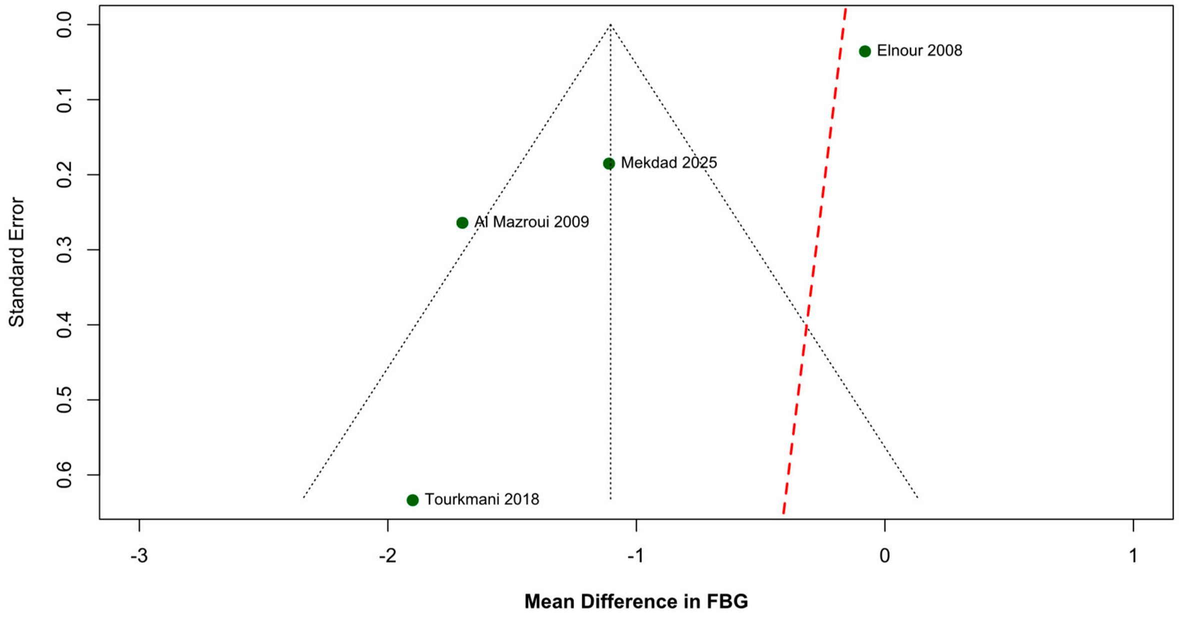

iii) SBP

(a) Sensitivity analysis for SBP

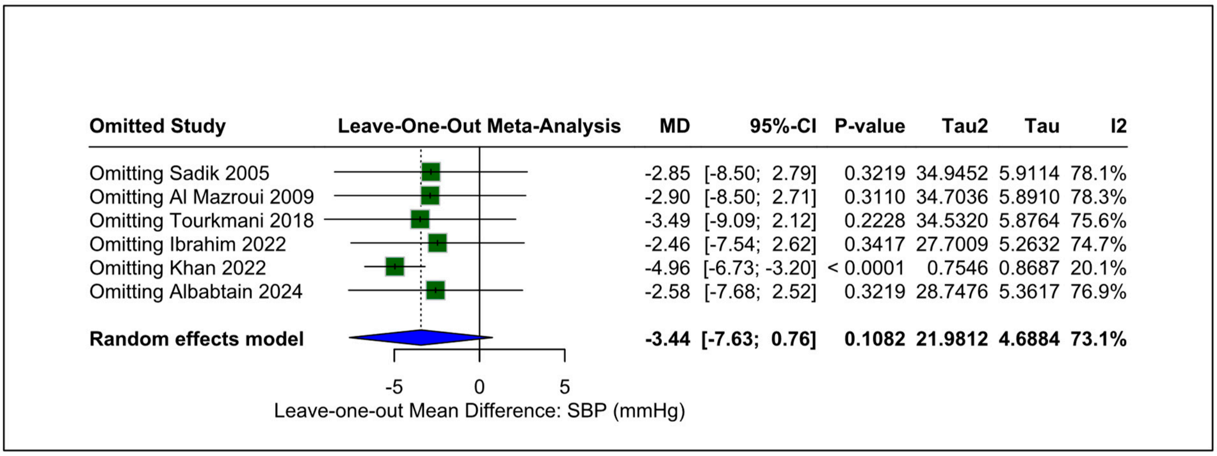

(b) Publication Bias for SBP

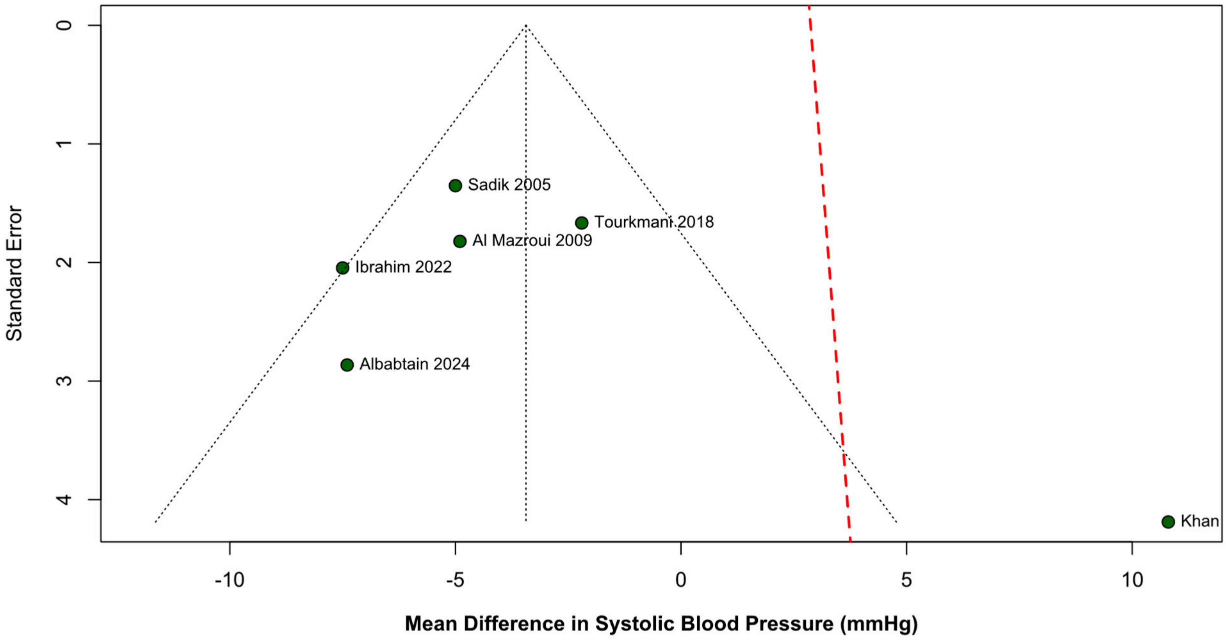

iv)DBP

(a) Sensitivity analysis for DBP

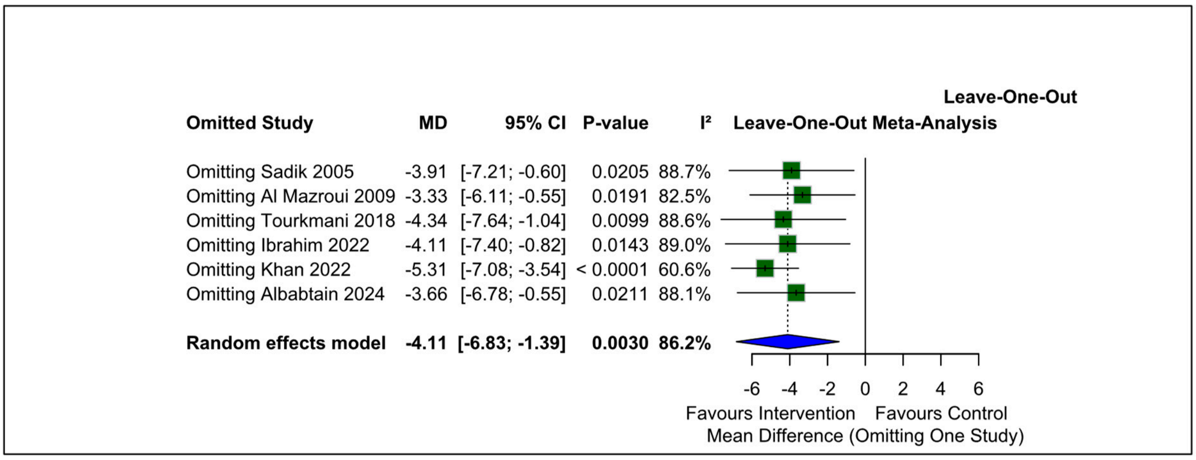

(b) Publication Bias for DBP

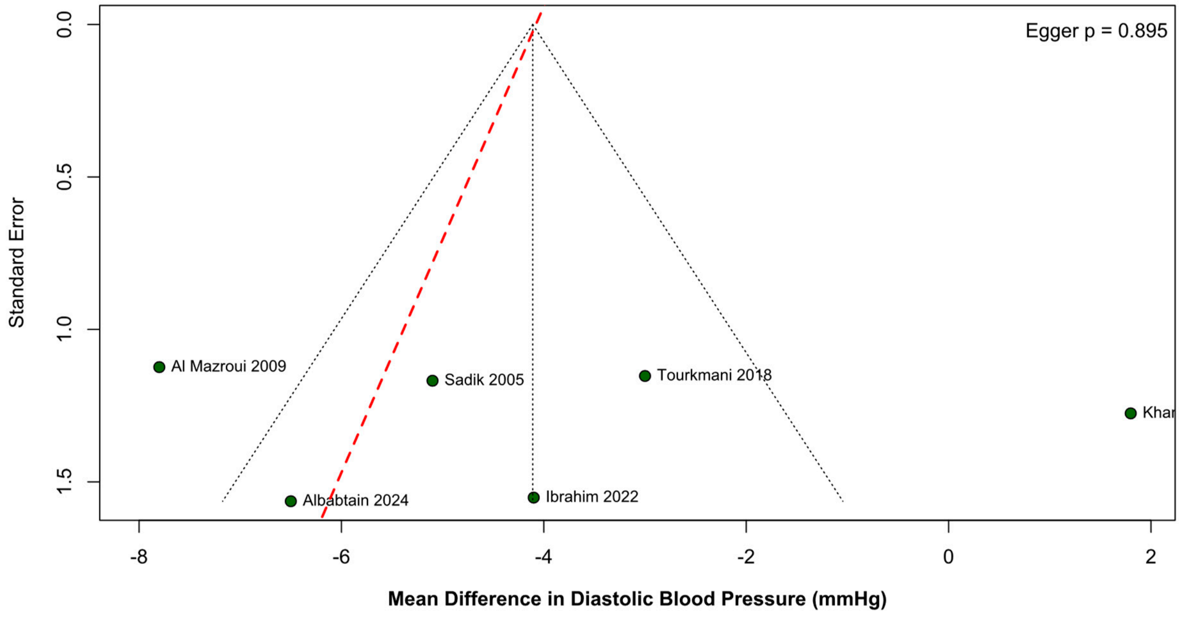

v) Lipid Profile

1. Total cholesterol

(a) Sensitivity analysis for Total Cholesterol

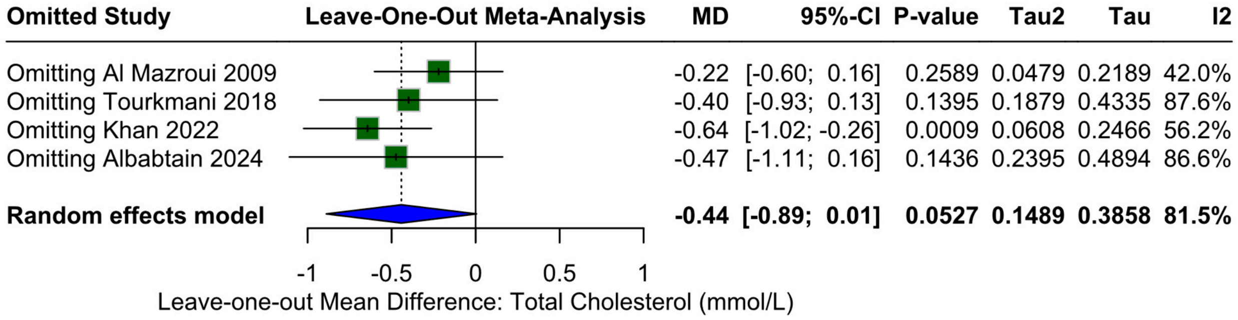

(b) Publication Bias for Total Cholesterol

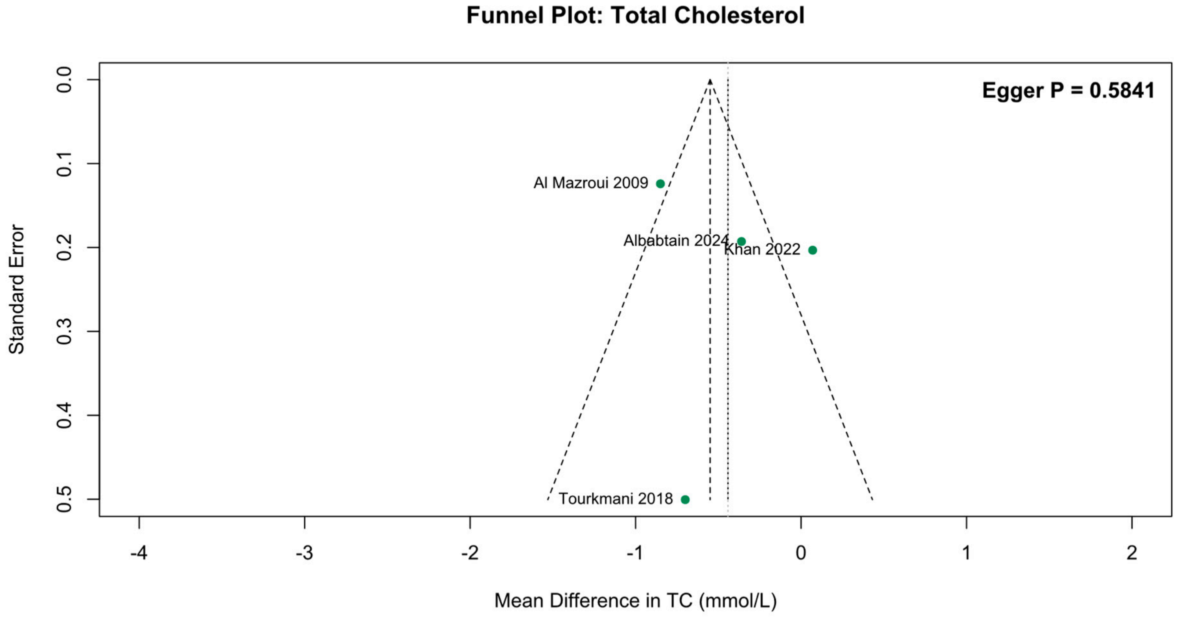

2. HDL Cholesterol

(a) Sensitivity analysis for HDL Cholesterol

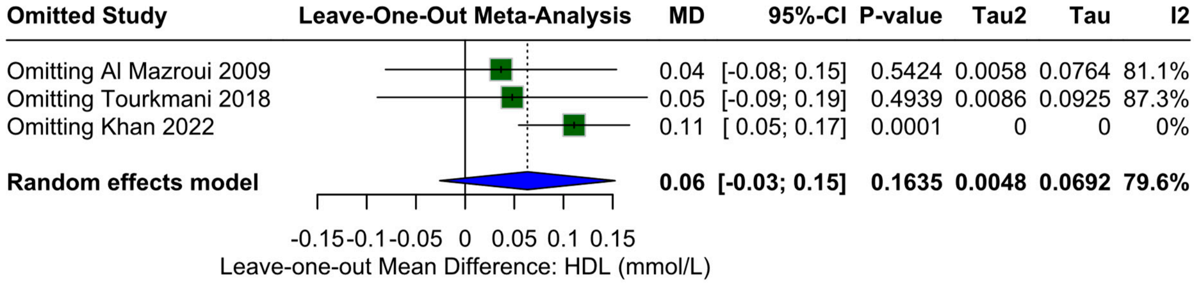

(b) Publication Bias for HDL Cholesterol

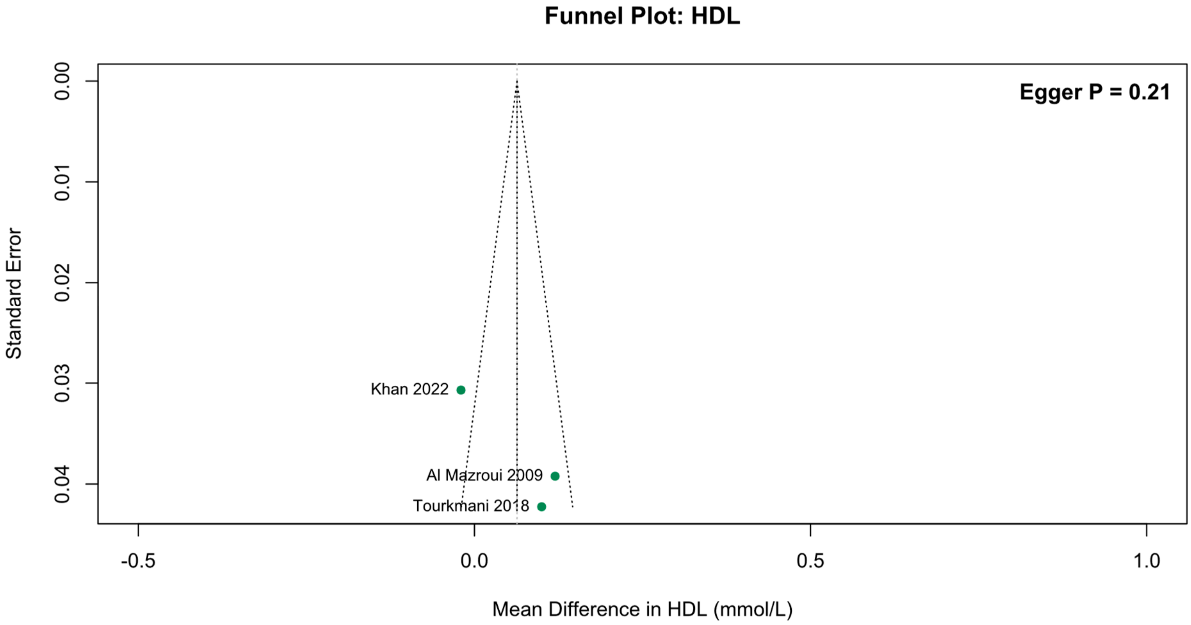

3. LDL Cholesterol

(a) Sensitivity analysis for LDL Cholesterol

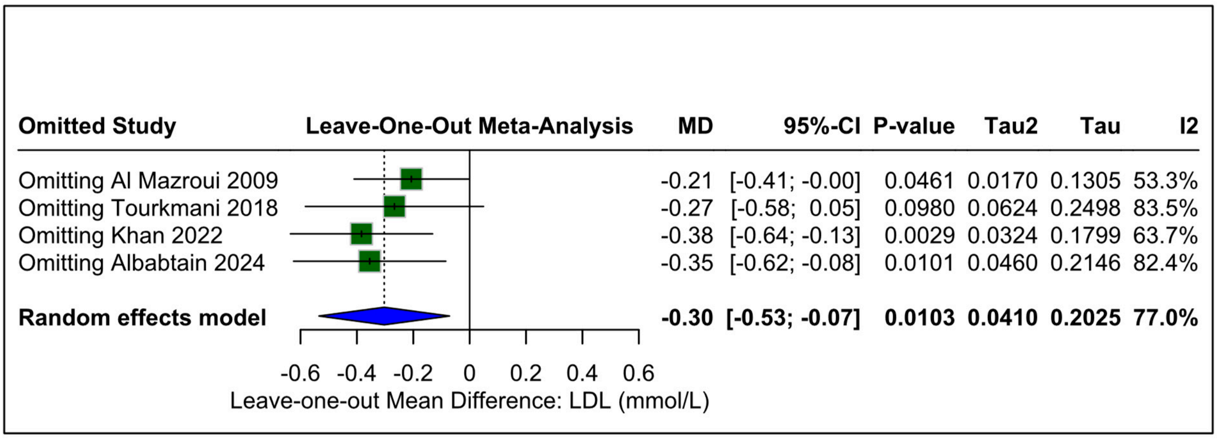

(b) Publication Bias for LDL Cholesterol

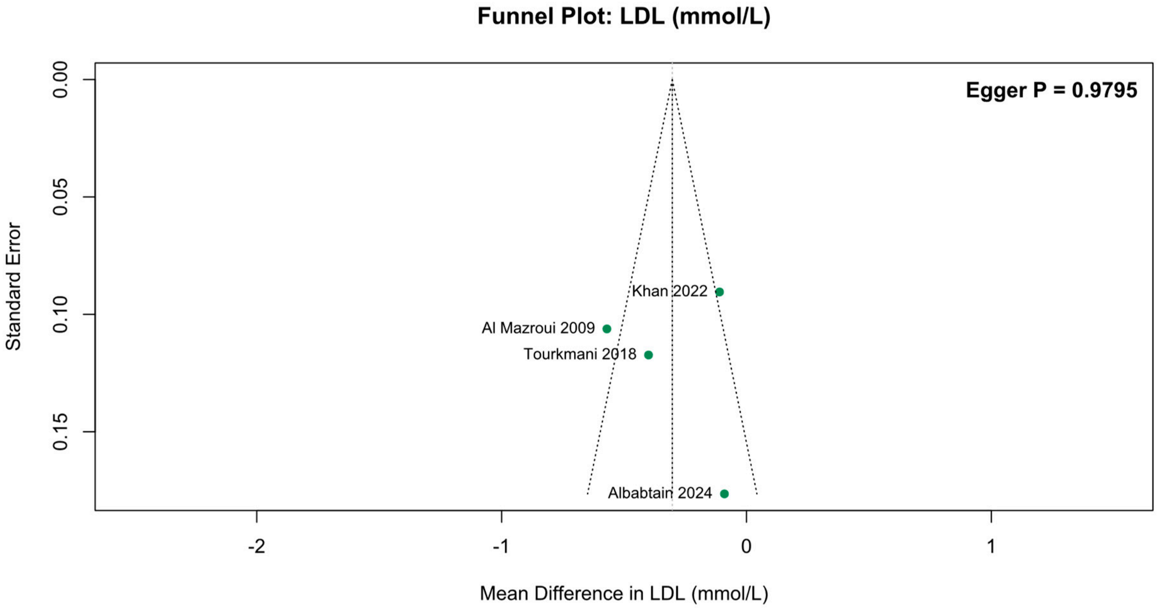

4. Triglycerides

(a) Sensitivity analysis for Triglycerides

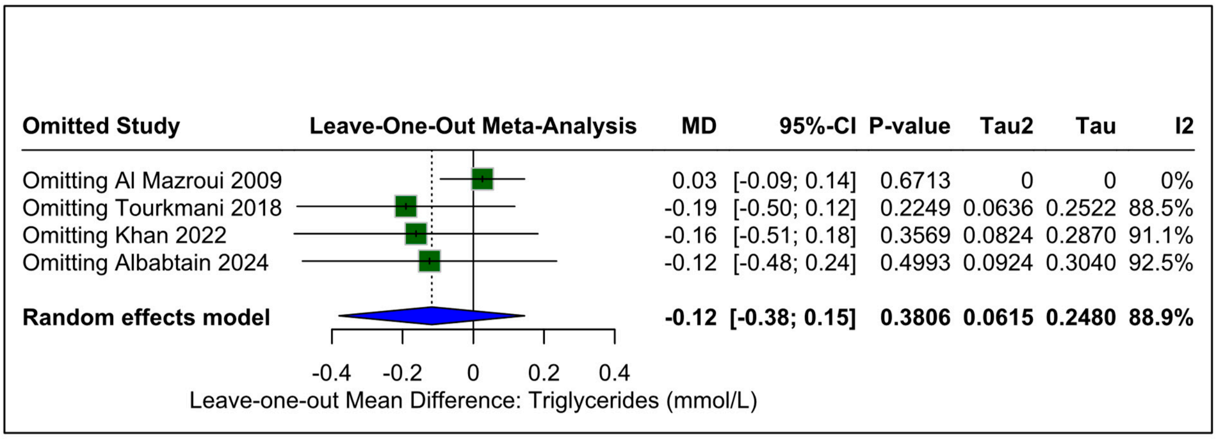

(b) Publication Bias for Triglycerides

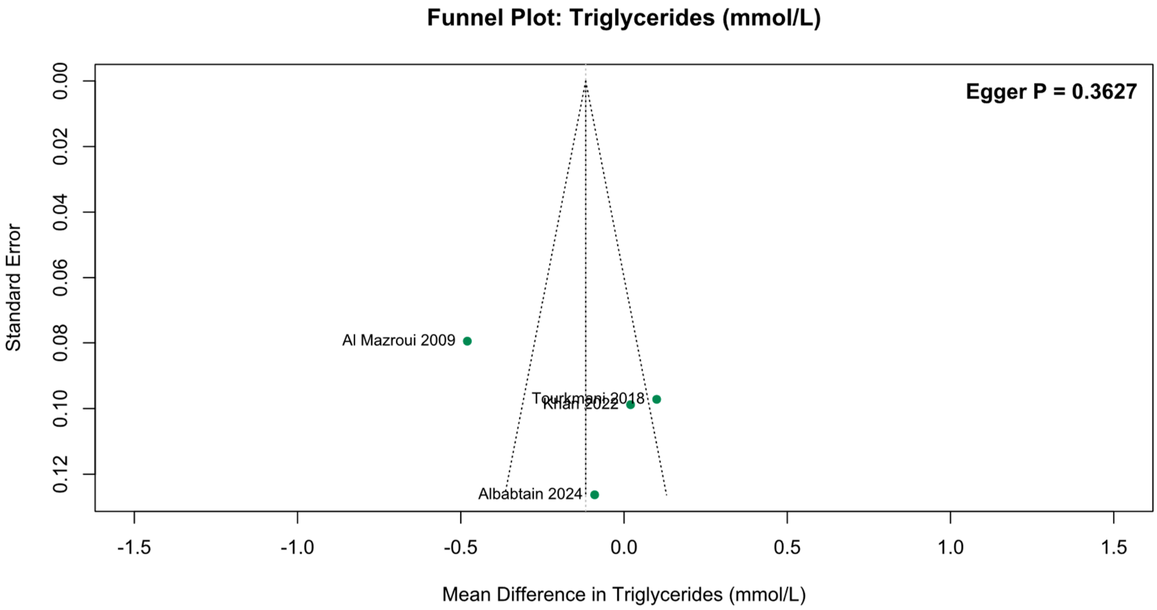

b) Quasi-Experimental Studies

i) Mortality

(a) Sensitivity analysis for Mortality

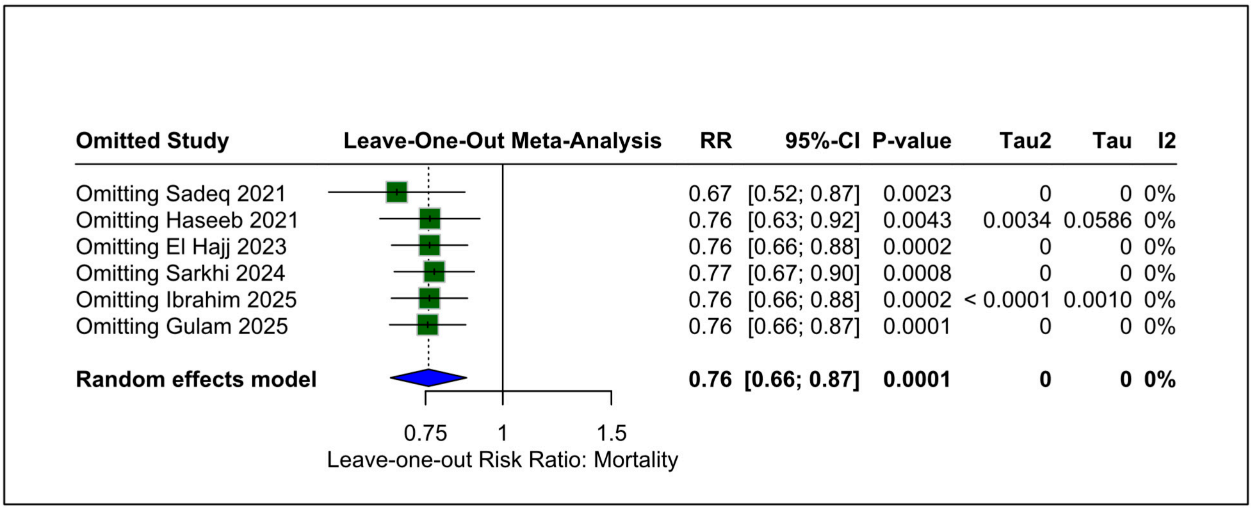

(b) Publication Bias for Mortality

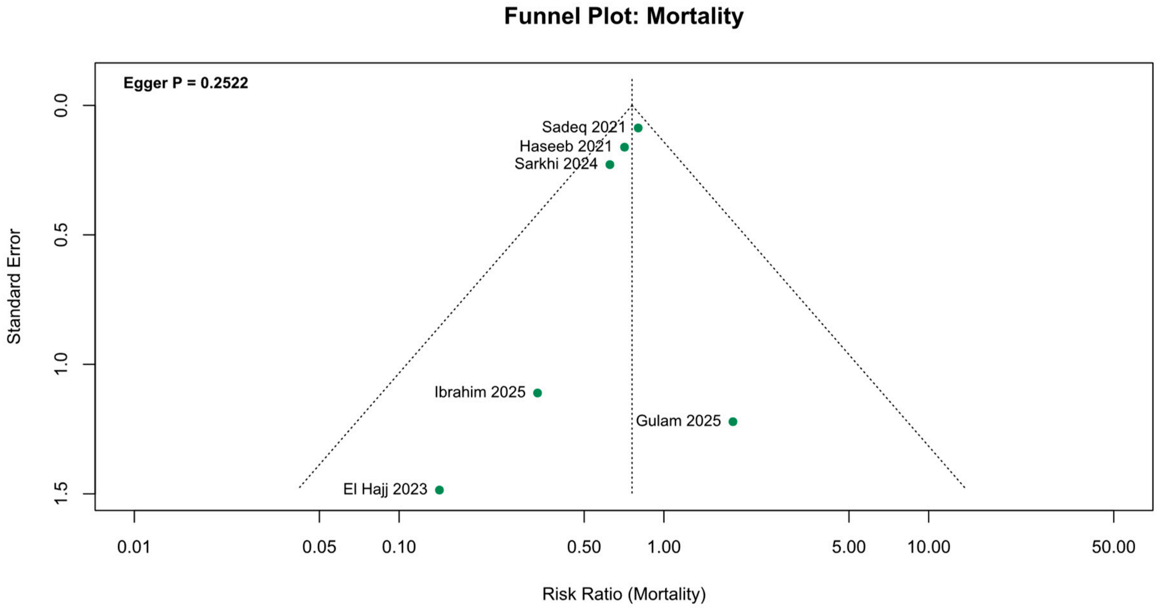

ii) Hospital Readmissions

(a) Sensitivity analysis for Hospital Readmissions

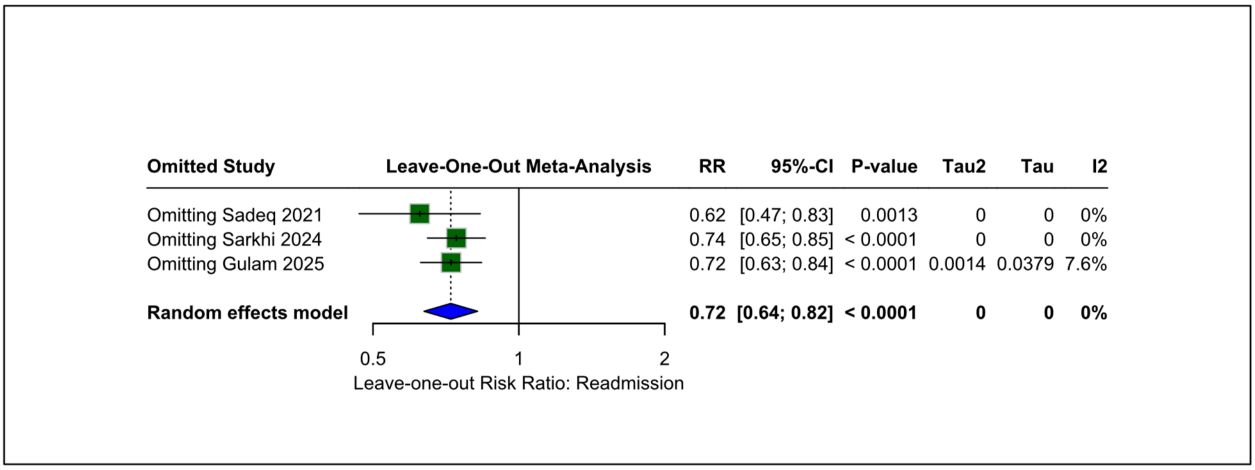

(b) Publication Bias for Hospital Readmissions

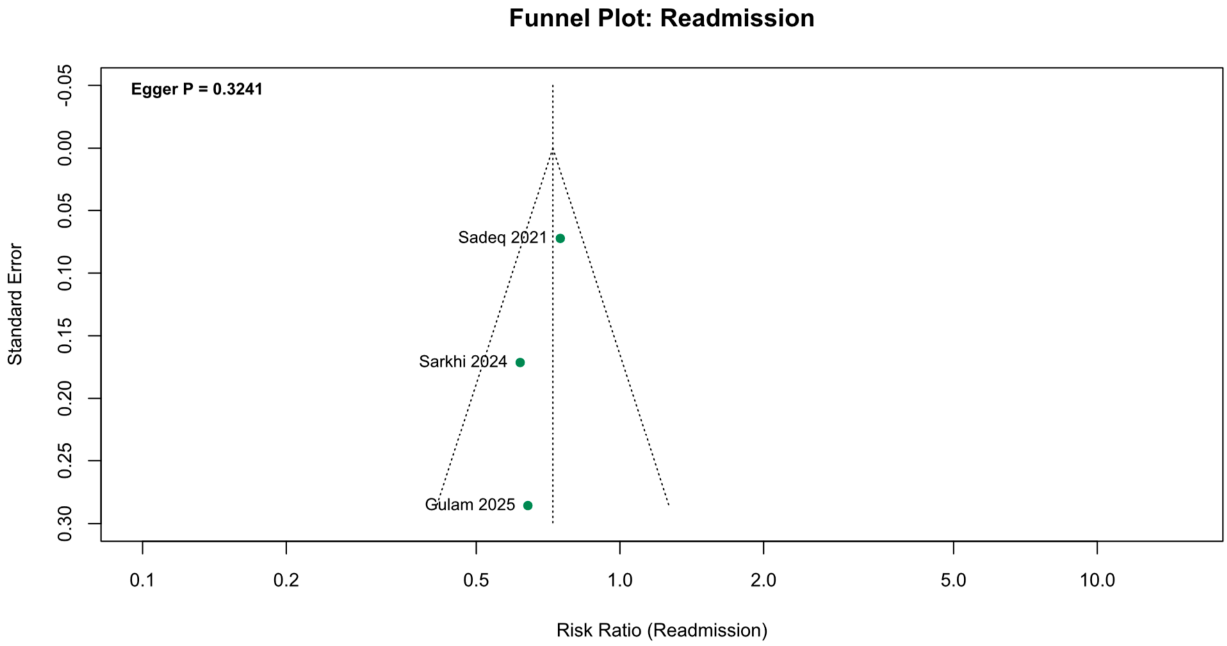

iii) Hospital length of stay (days)

(a) Sensitivity analysis for Hospital Length of stay (days)

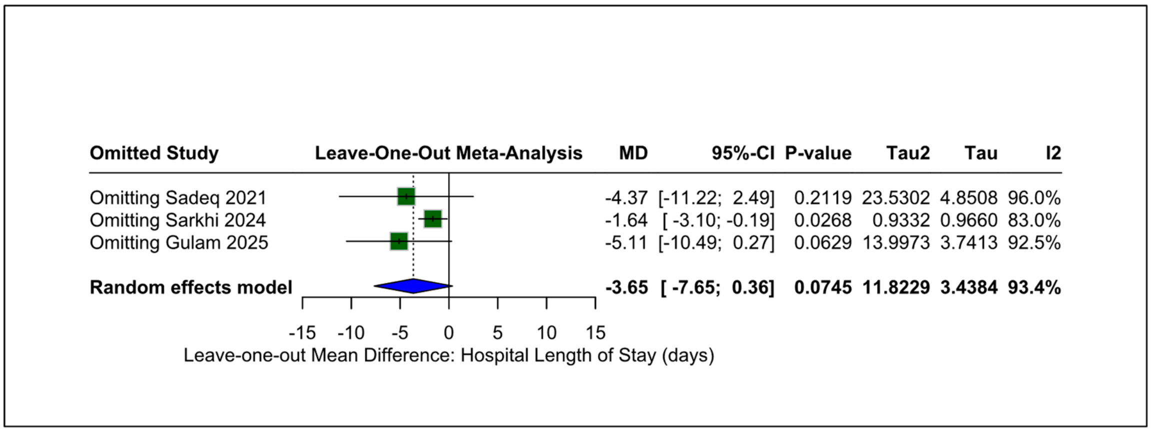

(b) Publication Bias for Hospital Length of stay (days)

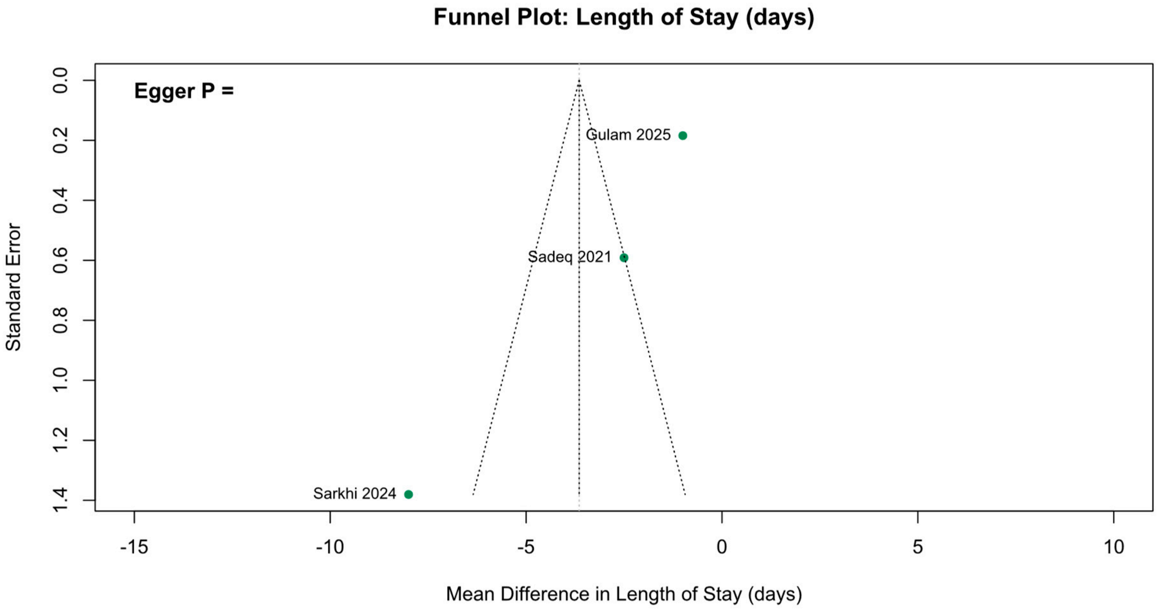

iv) ICU length of stay (days)

(a) Sensitivity analysis for ICU length of stay (days)

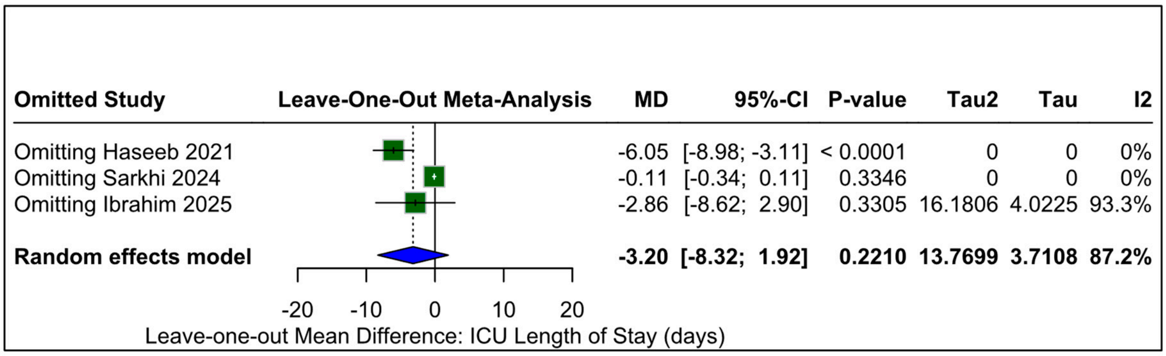

(b) Publication Bias for ICU length of stay (days)

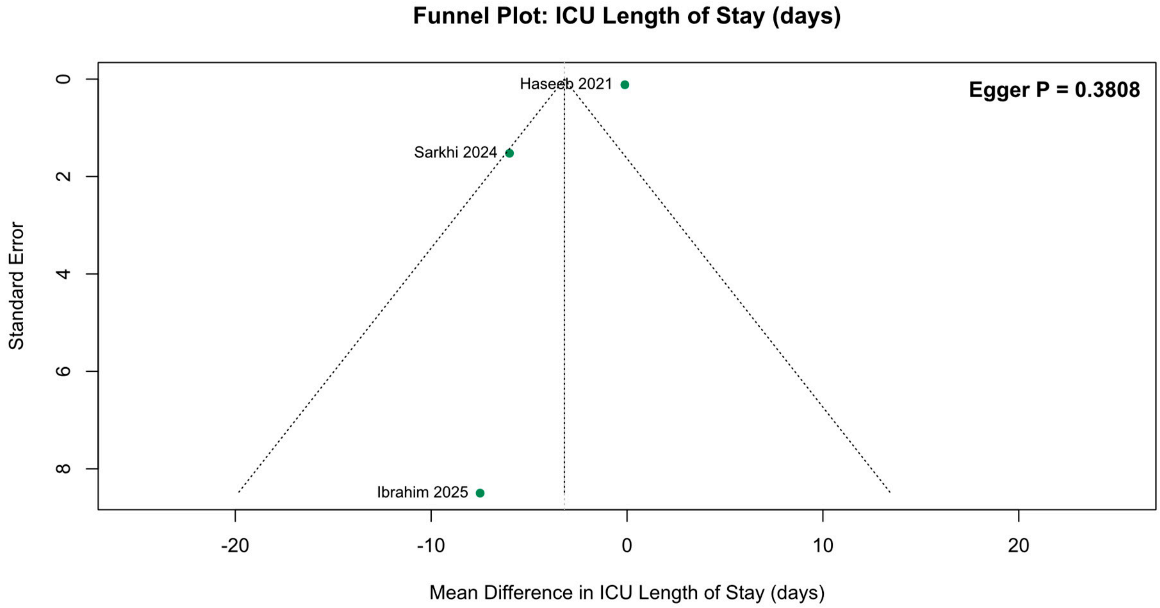

2) Humanistic outcomes

a) Randomized controlled Trials (RCTs)

i. Medication Adherence

(a) Sensitivity analysis for Medication Adherence

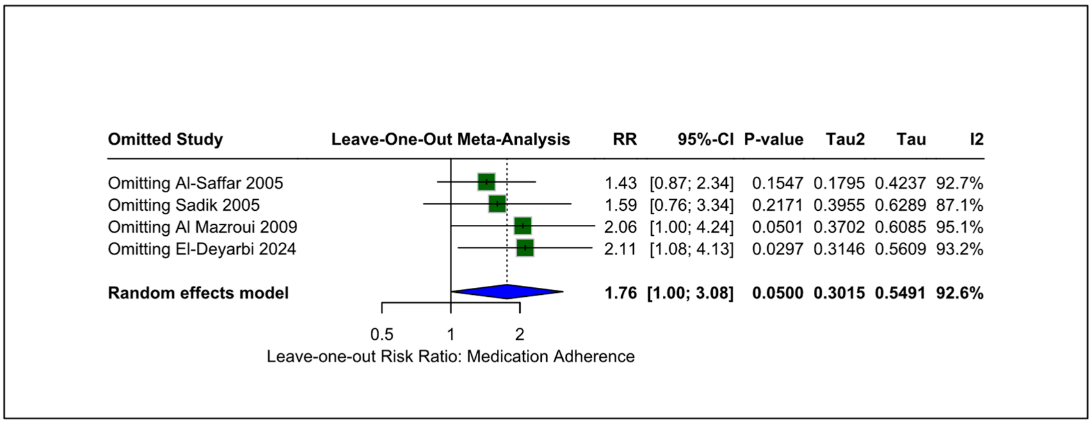

(b) Publication Bias for Medication Adherence

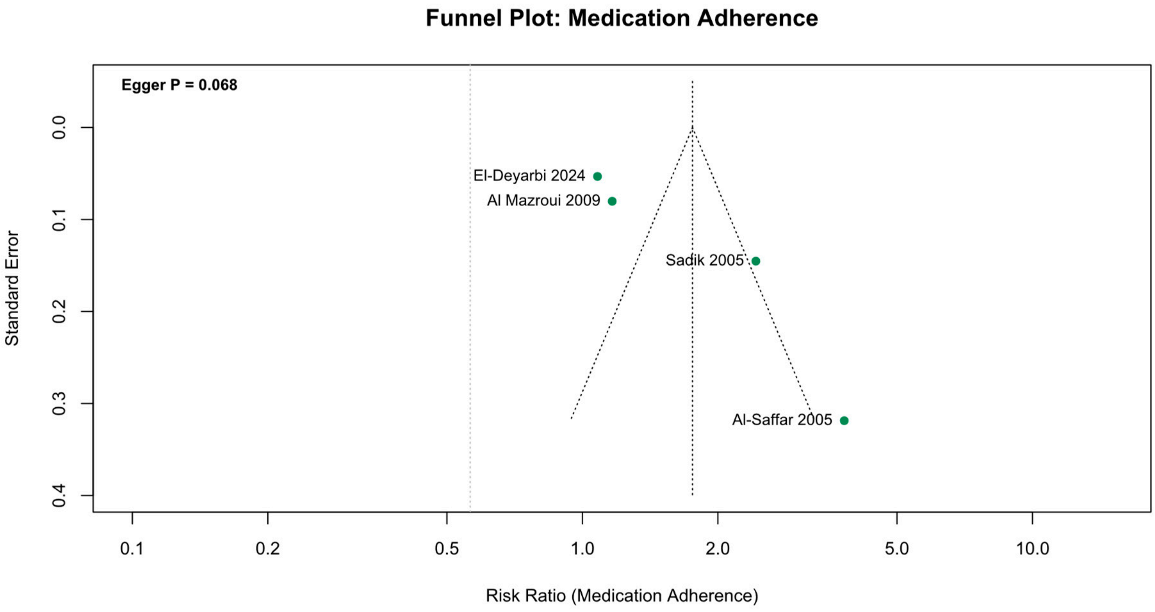

ii. Adequate Medication Knowledge

(a) Sensitivity analysis for Adequate Medication Knowledge

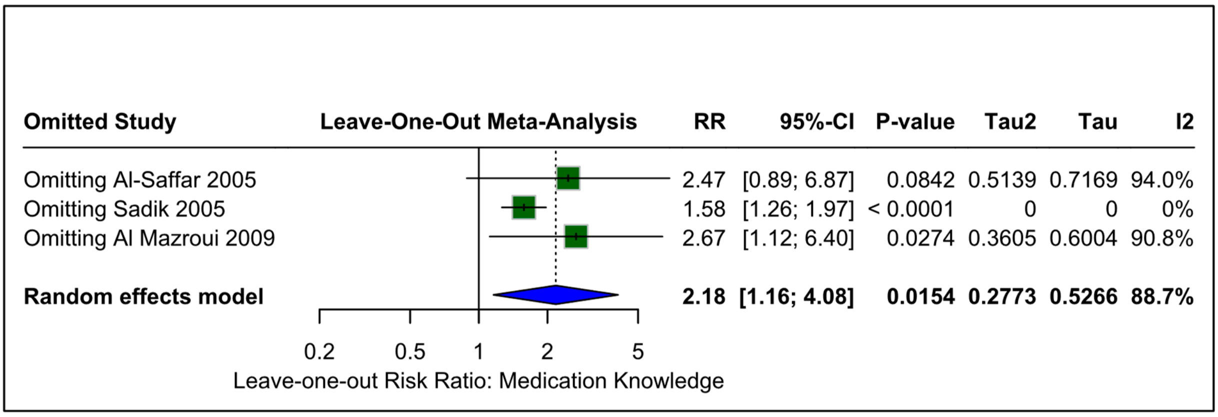

(b) Publication Bias for Adequate Medication Knowledge

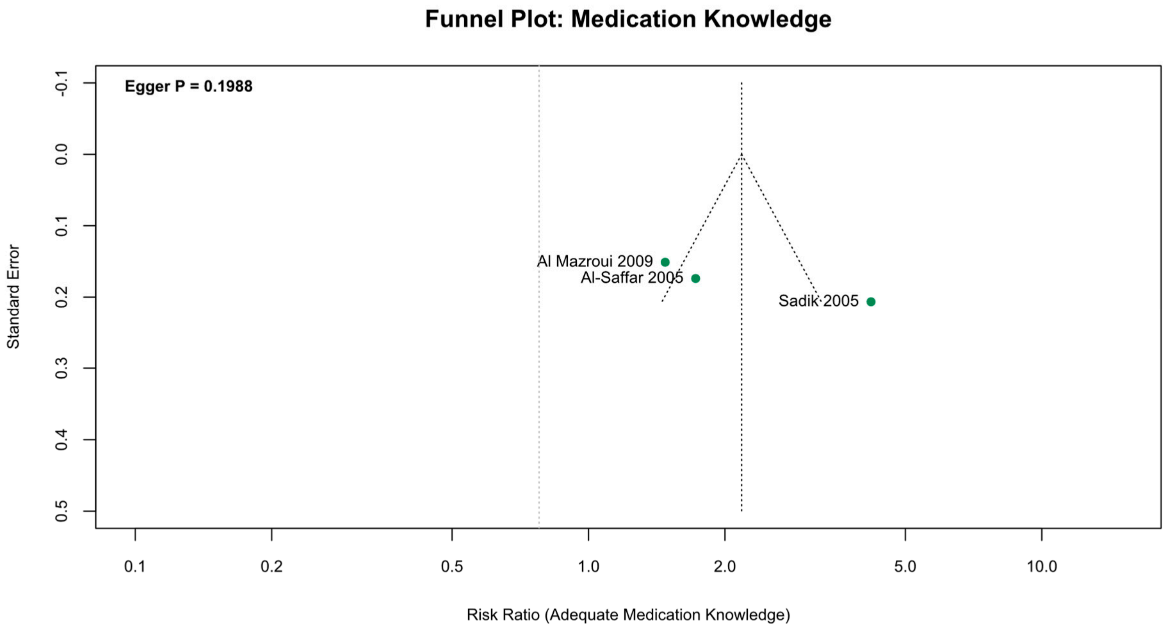

iii. SF 36 Quality of life domain outcomes

(a) Sensitivity analysis for SF 36 Quality of life domain outcomes

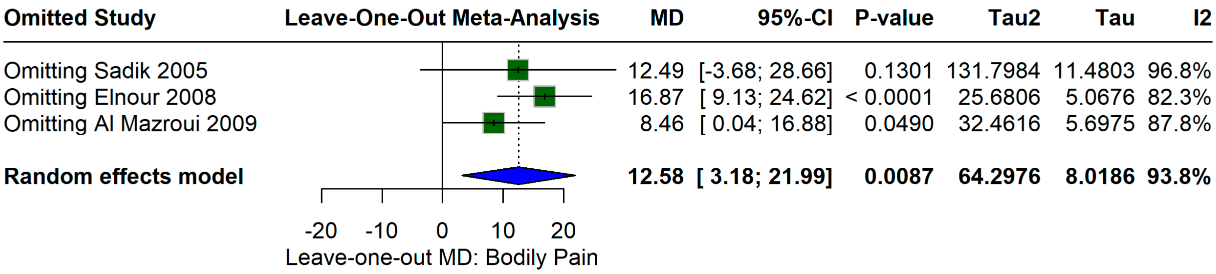

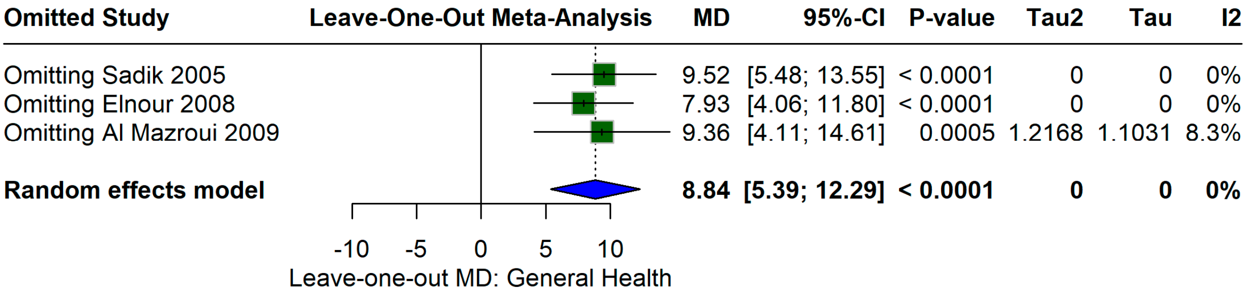

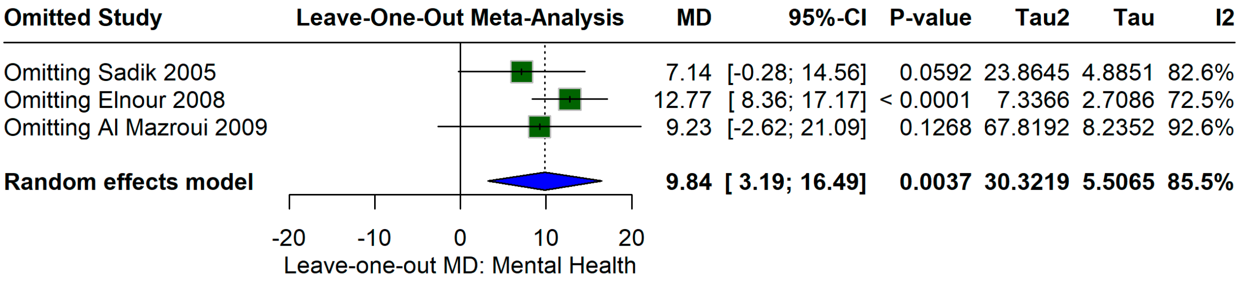

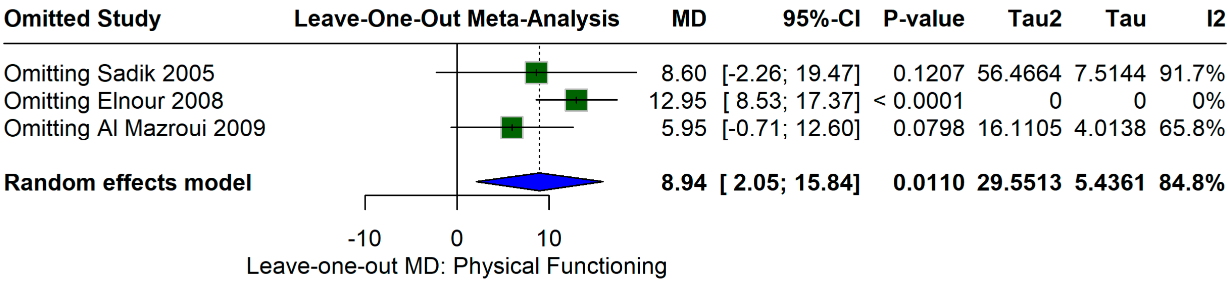

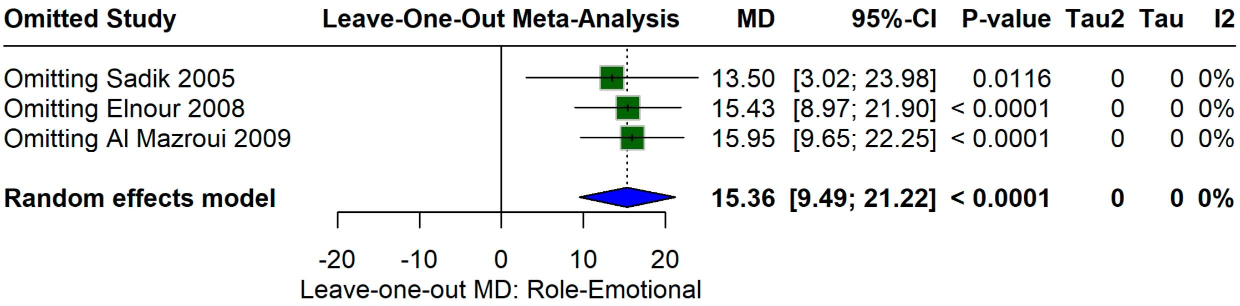

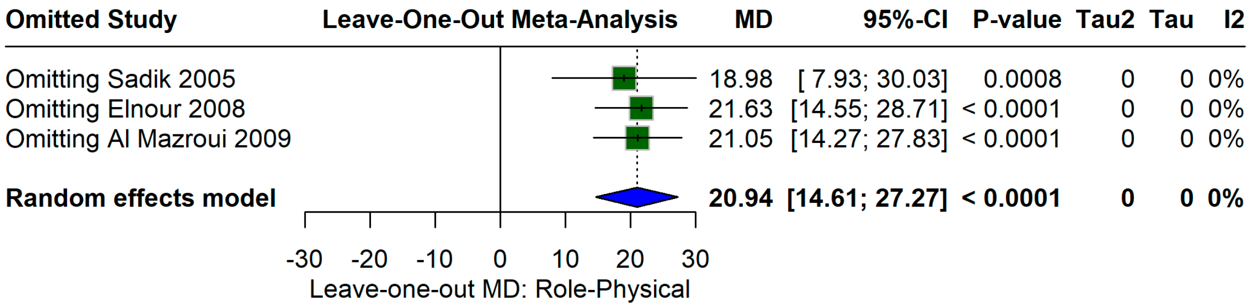
Omitted Study
Leave-One-Out Meta-Analysis
MD
95%-CI
P-value
Tau2
Tau
I2
Omitting Sadik 2005 18.98 [7.93; 30.03] 0.0008 0 0 0%
Omitting Elnour 2008 21.63 [14.55; 28.71] < 0.0001 0 0 0%
Omitting Al Mazroui 2009 21.05 [14.27; 27.83] < 0.0001 0 0 0%
Random effects model 20.94 [14.61; 27.27] < 0.0001 0 0 0%
-30
-20
-10
0
10
20
30
Leave-one-out MD: Role-Physical

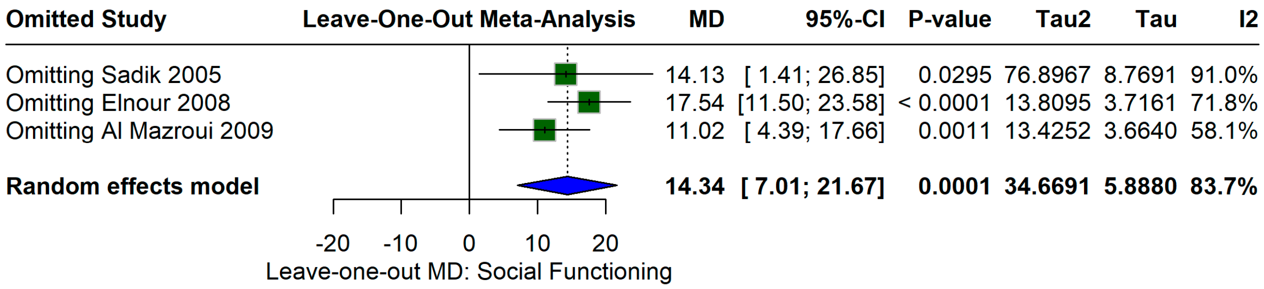
Omitted Study
Leave-One-Out Meta-Analysis
MD
95%-CI
P-value
Tau2
Tau
I2
Omitting Sadik 2005 14.13 [1.41; 26.85] 0.0295 76.8967 8.7691 91.0%
Omitting Elnour 2008 17.54 [11.50; 23.58] < 0.0001 13.8095 3.7161 71.8%
Omitting Al Mazroui 2009 11.02 [4.39; 17.66] 0.0011 13.4252 3.6640 58.1%
Random effects model 14.34 [7.01; 21.67] 0.0001 34.6691 5.8880 83.7%
-20
-10
0
10
20
Leave-one-out MD: Social Functioning

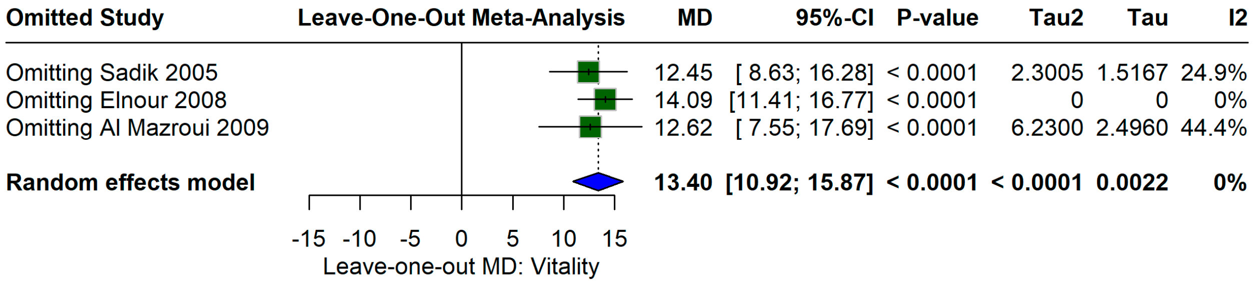
Omitted Study
Leave-One-Out Meta-Analysis
MD
95%-CI
P-value
Tau2
Tau
I2
Omitting Sadik 2005 12.45 [8.63; 16.28] < 0.0001 2.3005 1.5167 24.9%
Omitting Elnour 2008 14.09 [11.41; 16.77] < 0.0001 0 0 0%
Omitting Al Mazroui 2009 12.62 [7.55; 17.69] < 0.0001 6.2300 2.4960 44.4%
Random effects model 13.40 [10.92; 15.87] < 0.0001 < 0.0001 0.0022 0%
-15
-10
-5
0
5
10
15
Leave-one-out MD: Vitality

(b) Publication Bias for SF 36 Quality of life domain outcomes

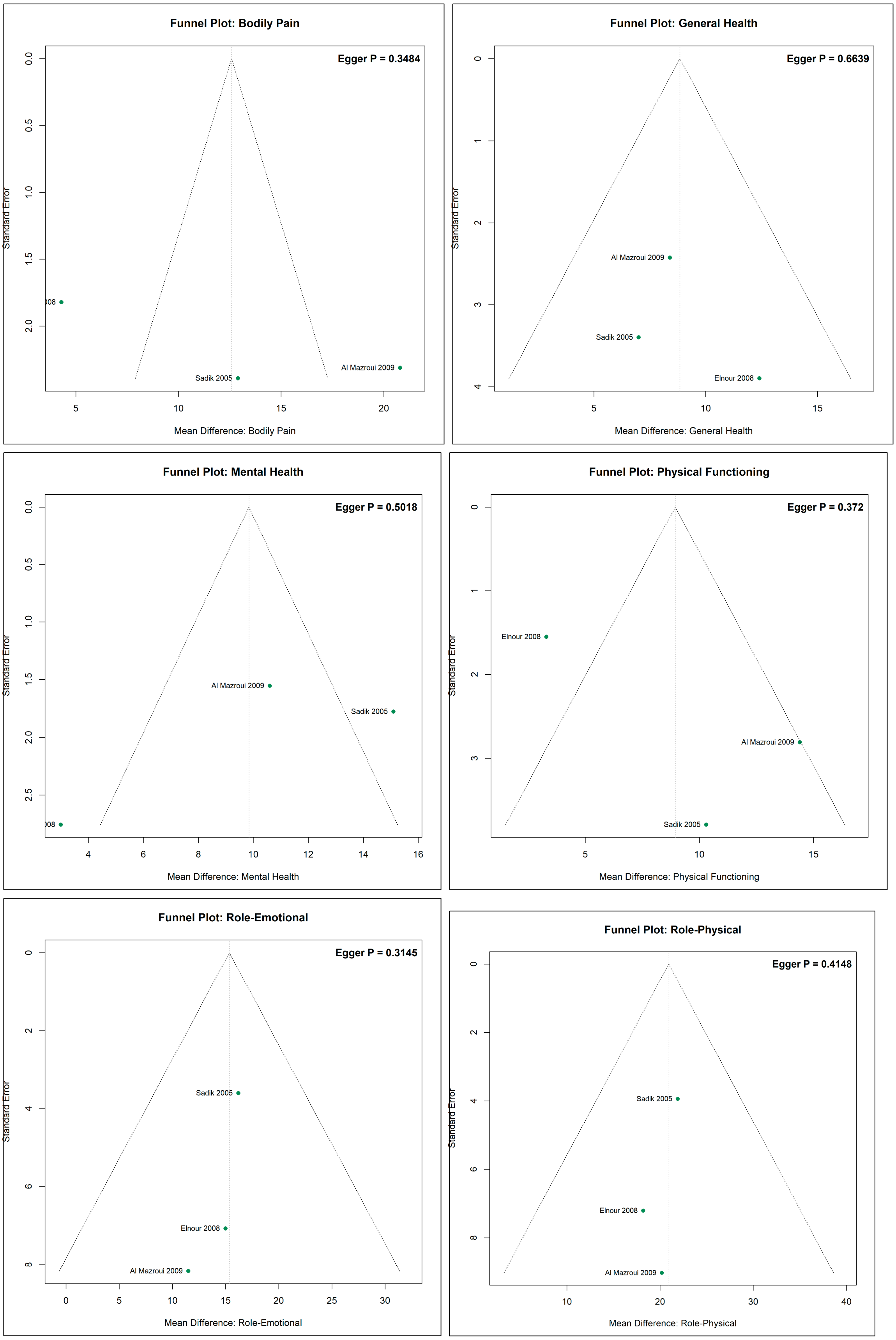

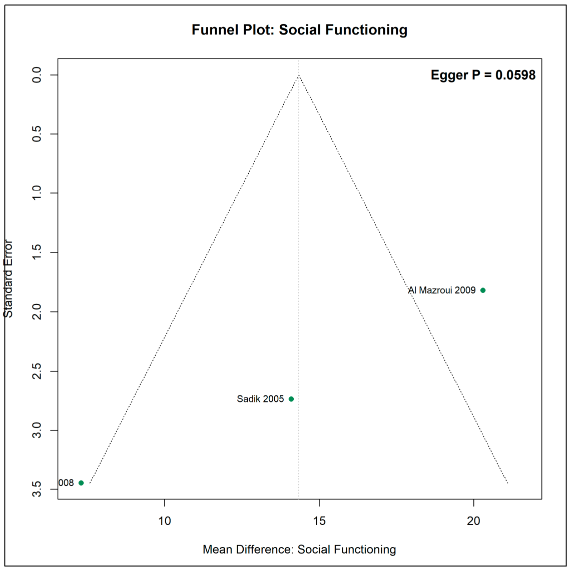

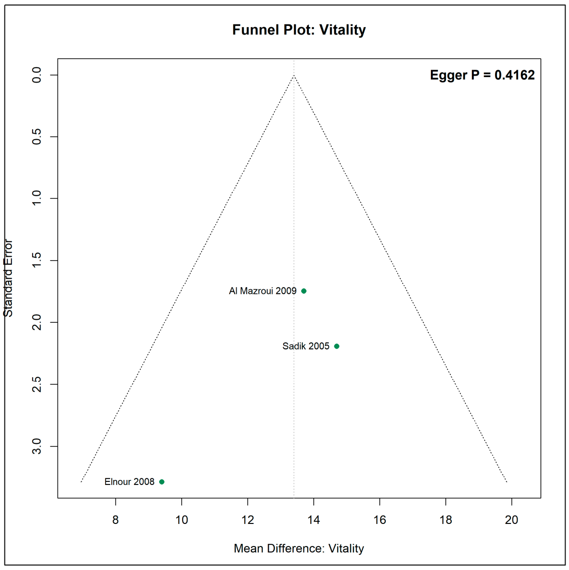

b) Quasi Experimental Studies

i. Antimicrobial Utilization (LOT/DOD, DDD)

(a) Sensitivity analysis for ICU length of stay (days)

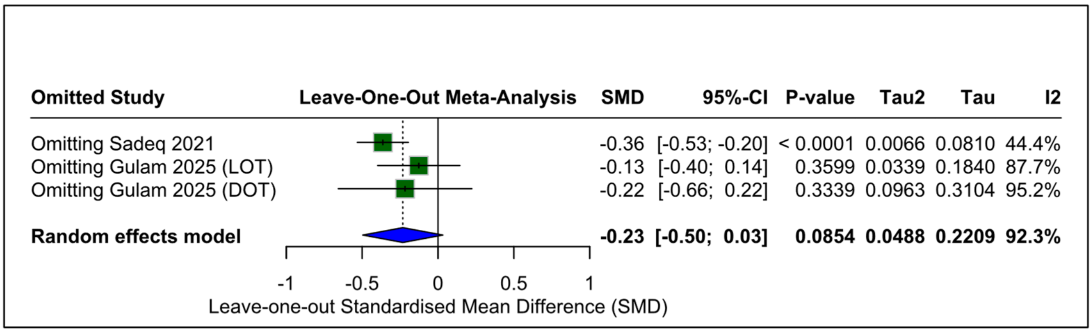

(b) Publication Bias for ICU length of stay (days)

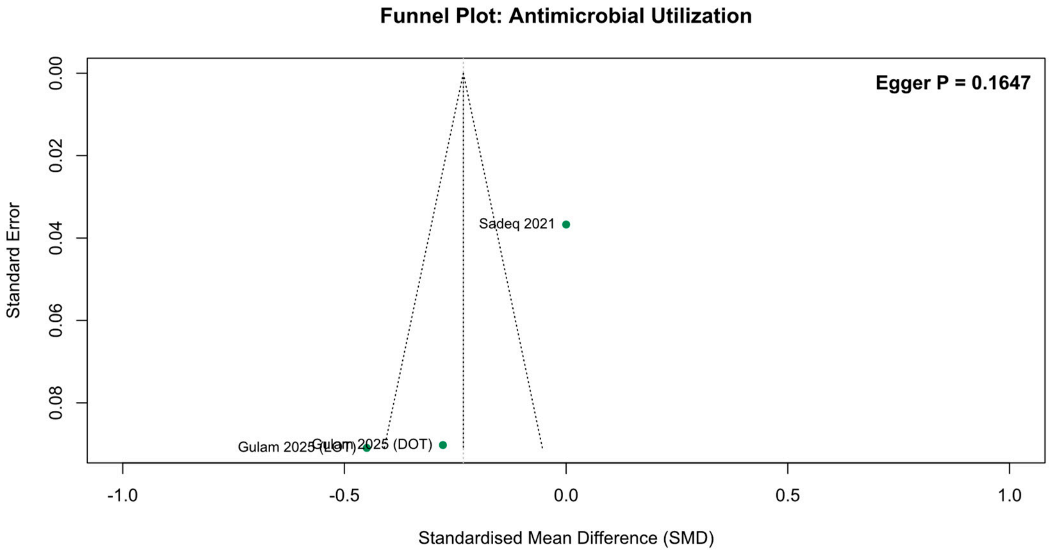

3) Economic outcomes

b) Quasi Experimental Studies

i. Health Care Resource Utilization

(a) Sensitivity analysis for Health Care Resource Utilization / Clinic Visit Frequency

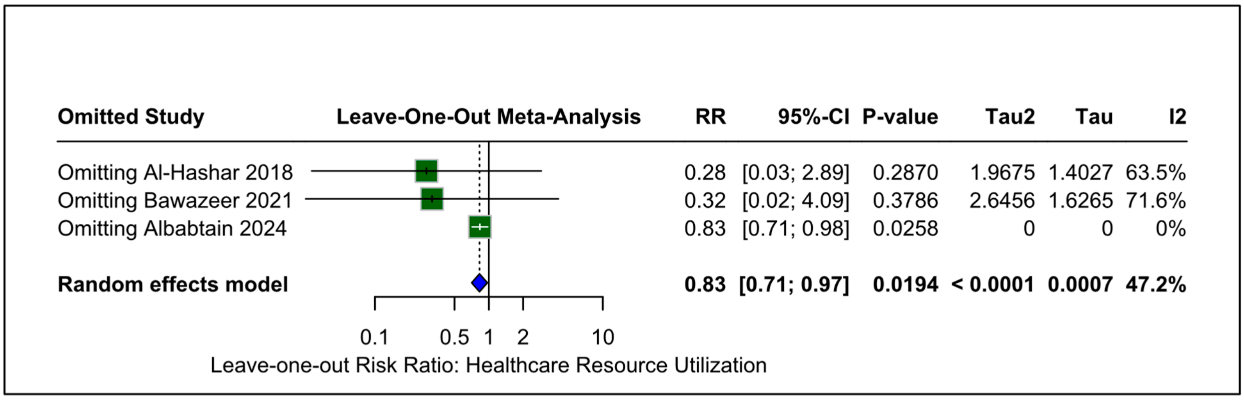

(b) Publication Bias for Health Care Resource Utilization / Clinic Visit Frequency

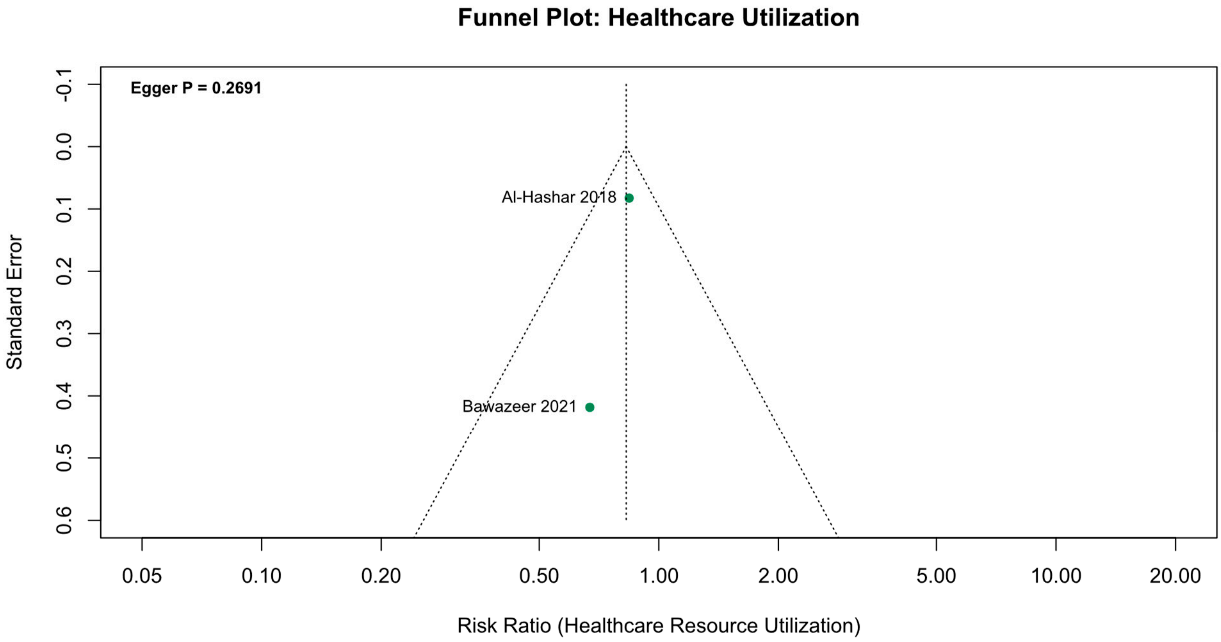

Supplement: Supplementary file 1 [file pharmacy-14-00102-s001.zip › Supplementary File S2.pdf]
