# Supplementary material for: Impact of Pharmacist-Led Interventions on Patient Outcomes in Gulf Cooperation Council Countries: A Systematic Review and Meta-Analysis
Source: Pharmacy (Basel). 2026 Jul 6;14(4):102. doi: 10.3390/pharmacy14040102 (PMC13415103; doi:10.3390/pharmacy14040102)
Supplement: Supplementary file 1 [file pharmacy-14-00102-s001.zip › Supplementary File S3.pdf]

Supplementary Data

Search Strategy, Key words and Mesh Terms

A:
("Pharmacists"[MeSH] OR
pharmacist*[tiab] OR
"clinical pharmacist"[tiab] OR
"pharmacist-led"[tiab] OR
"pharmacy-led"[tiab] OR
"clinical pharmacy"[tiab] OR
"pharmaceutical care"[tiab])
B:
(intervention*[tiab] OR
program*[tiab] OR
service*[tiab] OR
"medication therapy management"[tiab] OR
MTM[tiab] OR
"medication review"[tiab] OR
"medication reconciliation"[tiab] OR
counseling[tiab] OR
"patient education"[tiab] OR
"pharmaceutical care"[tiab] OR
"antimicrobial stewardship"[tiab] OR
discharge[tiab] OR
follow-up[tiab])
C:
("Saudi Arabia"[MeSH] OR
"United Arab Emirates"[MeSH] OR
"Oman"[MeSH] OR

"Qatar"[MeSH] OR
"Bahrain"[MeSH] OR
"Kuwait"[MeSH]
D:
(randomized[tiab] OR
randomised[tiab] OR
"randomized controlled trial"[pt] OR
"controlled trial"[tiab] OR
trial[tiab] OR
"quasi-experimental"[tiab] OR
"controlled before-after"[tiab] OR
"before and after"[tiab])

Table S1: Key words and Mesh Terms used to search the literature

History and Search Details

Download Delete

Search	Actions	Details	Query	Results	Time
#5	•••	>	Search: **#1 AND #2 AND #3 AND #4** Filters: **from 2000/1/1 - 2025/12/31**	24	15:47:27
#4	•••	>	Search: **(#3) AND (randomized[tiab] OR randomized[tiab] OR "randomized controlled trial"[pt] OR "controlled trial"[tiab] OR trial[tiab] OR "quasi-experimental"[tiab] OR "controlled before-after"[tiab] OR "before and after"[tiab])** Filters: **from 2000/1/1 - 2025/12/31**	24	15:44:06
#3	•••	>	Search: **(#2) AND ("Saudi Arabia"[MeSH] OR "United Arab Emirates"[MeSH] OR "Oman"[MeSH] OR "Qatar"[MeSH] OR "Bahrain"[MeSH] OR "Kuwait"[MeSH])** Filters: **from 2000/1/1 - 2025/12/31**	274	15:33:36
#2	•••	>	Search: **(#1) AND ((intervention*[tiab] OR program*[tiab] OR service*[tiab] OR "medication therapy management"[tiab] OR MTM[tiab] OR "medication review"[tiab] OR "medication reconciliation"[tiab] OR counseling[tiab] OR "patient education"[tiab] OR "pharmaceutical care"[tiab] OR "antimicrobial stewardship"[tiab] OR discharge[tiab] OR follow-up[tiab]))** Filters: **from 2000/1/1 - 2025/12/31**	28,739	15:12:53
#1	•••	>	Search: **("Pharmacists"[MeSH] OR pharmacist*[tiab] OR "clinical pharmacist"[tiab] OR "pharmacist-led"[tiab] OR "pharmacy-led"[tiab] OR "clinical pharmacy"[tiab] OR "pharmaceutical care"[tiab])** Filters: **from 2000/1/1 - 2025/12/31**	48,448	15:11:33

Showing 1 to 5 of 5 entries

Table S2: Key words and Mesh Terms used to search the literature for PubMed/MEDLINE (Example)

Risk of Bias Assessment

Table S3: Risk of Bias in Randomized Controlled Trials

S. No	Author (Year)	Randomization	Deviations from Intervention	Missing Data	Outcome Measurement	Selective Reporting	Overall Risk
1	Sadik et al., 2005	Low	High	Low	Low	Low	Moderate
2	Al-Saffar et al., 2005	Low	High	Moderate	Low	Low	Moderate
3	Elnour et al., 2008	Low	High	Low	Low	Low	Moderate
4	Al Mazroui et al., 2009	Low	Low	Low	Low	Low	Low
5	El Hajj et al., 2017	Low	Low	High	Low	Low	Moderate
6	Al-Hashar et al., 2018	Low	Low	Low	Low	Low	Low
7	Tourkmani et al., 2018	Low	Low	Low	Low	Low	Low
8	Bawazeer et al., 2021	Low	High	Low	Low	Low	Moderate
9	Khan et al., 2022	Low	High	Low	Low	Low	Moderate
10	Ibrahim et al., 2022	Low	High	Low	Low	Low	Moderate
11	El-Deyarbi et al., 2024	Low	Low	High	Low	Low	Moderate
12	Albabtain et al., 2024	Low	Low	Low	Low	Low	Low
13	Mekdad et al., 2025	Low	Moderate	Low	Low	Moderate	Moderate

Table S4: Risk of Bias in Quasi experimental studies

S. No.	Author (Year)	Confounding	Participant Selection	Intervention Classification	Deviations From Intervention	Missing Data	Outcome Measurement	Selective Reporting	Overall Risk of Bias
1	AlAjmi et al., 2017	Moderate	Moderate	Low	Low	Low	Low	Low	Moderate
2	Sadeq et al. 2021	Moderate	Moderate	Low	Low	Low	Low	Low	Moderate
3	Haseeb et al., 2021	Moderate	Moderate	Low	Low	Low	Low	Low	Moderate
4	El Hajj et al. 2023	Moderate	Moderate	Low	Low	Low	Low	Low	Moderate
5	Sarkhi et al. 2024	Moderate	Moderate	Low	Low	Low	Low	Low	Moderate
6	Ibrahim et al. 2025	Moderate	Moderate	Low	Low	Low	Low	Low	Moderate
7	Gulam et al. 2025	Moderate	Moderate	Low	Low	Low	Low	Low	Moderate

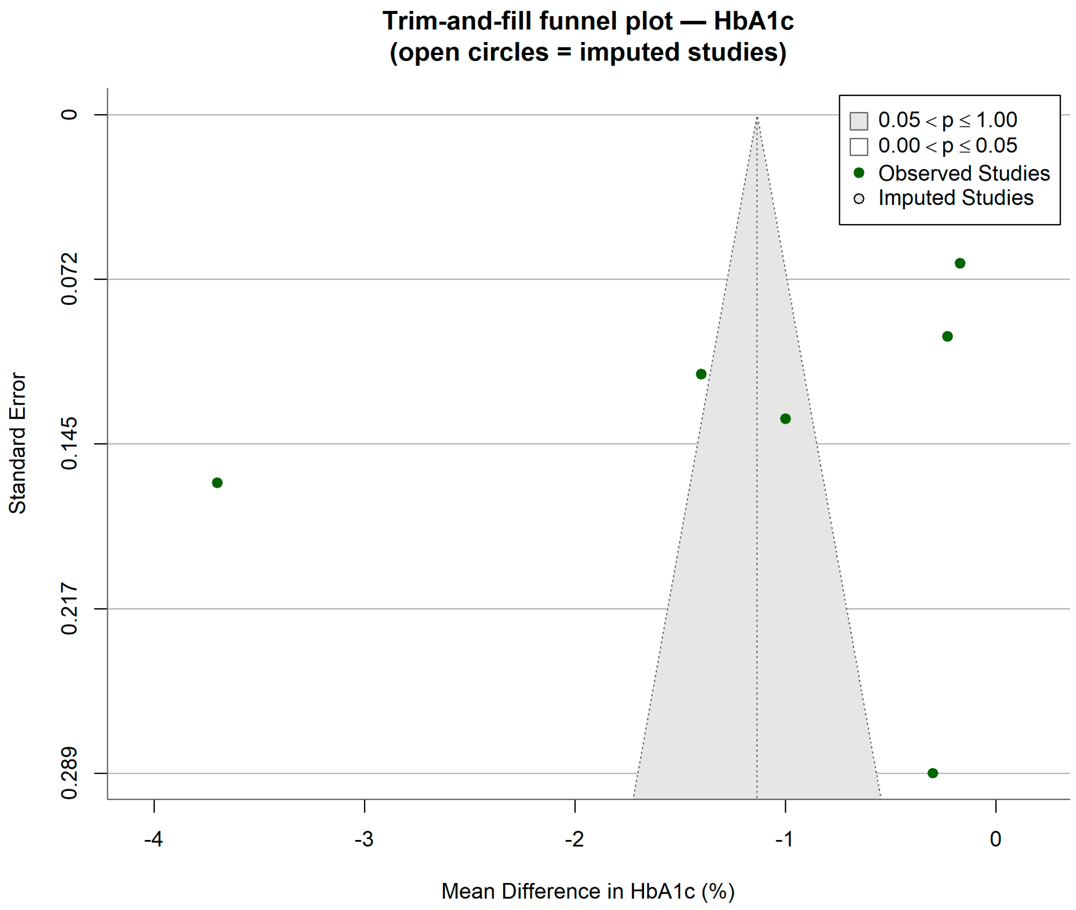

Supplementary Figure S1: Trim and Fill analysis of HbA1c estimating studies.

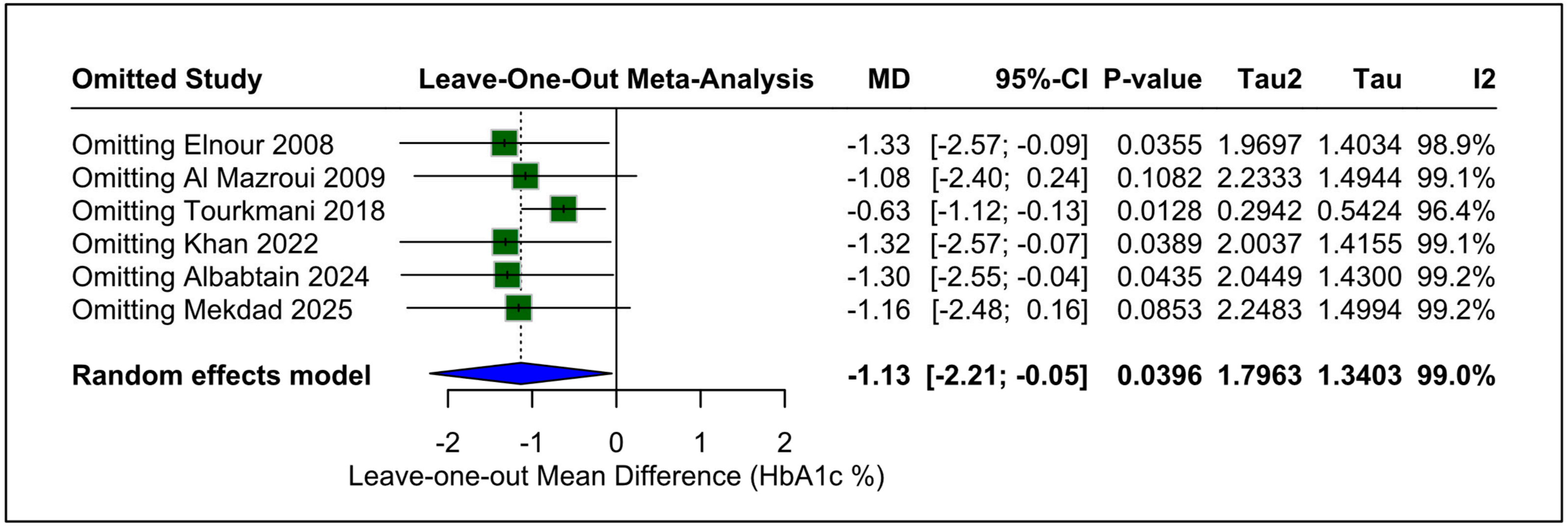

Supplementary Figure S2. Sensitivity analysis for HbA1c

Table S5: Detailed Statistical Findings for Non-Poolable Outcomes

Domain	Outcome	Finding	P-value	Citation from pooled papers
**Clinical**	BMI Reduction	Reduced BMI	P<.005	23
	10-year CHD Risk	10.6%→7.7%	P<.001	23
	Target BP Attainment	<130/80 mmHg: 33.6% vs 25.4%	P=.0213	23
	Target HbA1c Attainment	<7%: 45.4% vs 30.3%	P=.0213	23
	Weight Gain	Weight gain 3.7%	P<.001	24
	Insulin Dose	1.3 vs 0.6 U/kg (97.4% vs 63.2%)	P<.001	24
	Chronic Insulin Use	37.4% vs 22.7%	P=.047	33
	Pre-eclampsia	5.1% vs 16.7%	P=.014	33
	Caesarean Rate	7.1% vs 18.2%	P=.028	33
	Hypoglycemia (Study 1)	Severe episodes 2.24→1.20	P<.001	25
	Hypoglycemia (Study 2)	Non-significant	P=.65	28
	Anticoagulation INR	INR difference	P=.088	35
	Anticoagulation TTR	Days in therapeutic range	P=.46	35
	Functional Capacity	Improved 2-min walk & pulse	P<.05	30
	Renal Function	Serum creatinine −9.6%	P=.011	27
	Renal ACR	Non-significant trend	—	27
**Humanistic**	Objective Adherence	PDC>75%: 60.7% vs 60.0% vs 50.0%	P=.156	42
	Self-reported Adherence	MMAS-8: 6.70 vs 5.83	P=.024	36
	Lifestyle Scores	Increased	P<.01–<.001	23,25,29,30
	Knowledge Scores	Increased	P<.01–<.001	23,25,29,30
	Patient Satisfaction	PSPS improved	P=.00001	27,35
	Diabetes Distress	DDQ odds reduced 93.4%	P<.001	27,35
**Process**	Digital App Use	45.7% vs 21.4%	P<.01	26
	Digital Booklet Use	27.6% vs 1%	P<.01	26
	Medication Errors	Median 1 vs 7	P<.001	41
	Guideline Concordance	75% vs 10%	P<.001	41

	Antibiotic De-escalation	62.0% vs 40.6%	P<.001	39
	Preventable ADEs (non-adherence)	Associated	P=.049	34
	Preventable ADEs (Discrepancies)	Associated	P=.037	34
**Economic**	Clinic Visits	11.9 vs 5.1	P<.001	24,32
	Clinic Attendance	OR 2.1–3.2	P<.005	24,32
	Length of Stay	9.3 vs 10.2 days	—	37,41
	Cost Savings	£32,670 vs £56,580	—	37,41
	Antimicrobial Costs (Linezolid included)	Increased	P=.678	38
	Antimicrobial Costs (Linezolid excluded)	37% reduction	P=.008	38

1- Physical Functioning

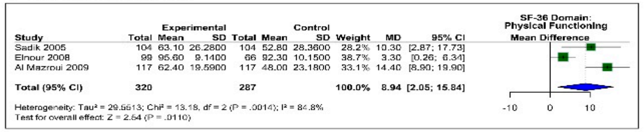

2- Role-Physical

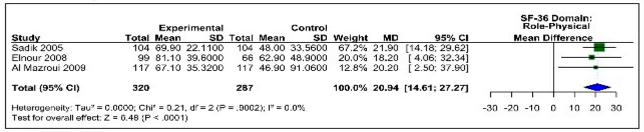

3- Bodily Pain

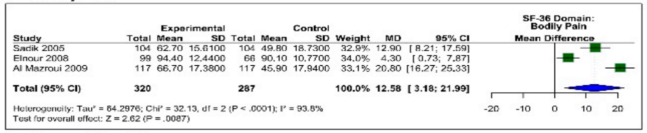

4- General Health

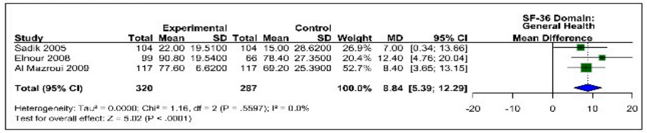

5- Vitality

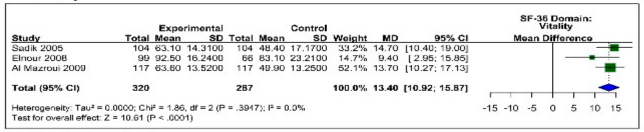

6- Social Functioning

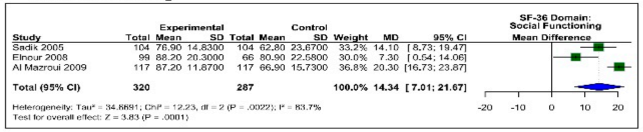

7- Role-Emotional

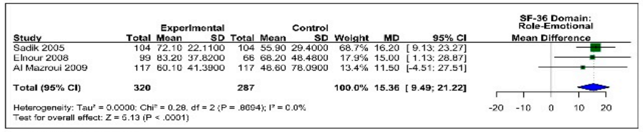

8- Mental Health

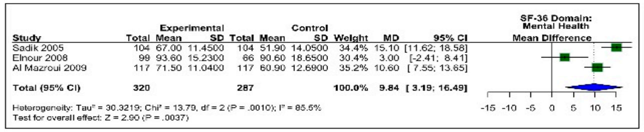

Supplementary Figure S3: SF-36 Domain health come outcomes forest plots.
